# The IronChip evaluation package: a package of perl modules for robust analysis of custom microarrays

**DOI:** 10.1186/1471-2105-11-112

**Published:** 2010-03-01

**Authors:** Yevhen Vainshtein, Mayka Sanchez, Alvis Brazma, Matthias W Hentze, Thomas Dandekar, Martina U Muckenthaler

**Affiliations:** 1 MMPU, Department of Pediatric Oncology, Hematology and Immunology, University of Heidelberg D-69120 Heidelberg, Germany; 2 European Molecular Biology Laboratory, D-69117 Heidelberg, Germany; 3 Department of Bioinformatics, University of Wurzburg, Biozentrum, Am Hubland, D-97074 Wurzburg, Germany; 4 IMPPC, Institute of Predictive and Personalized Medicine of Cancer, Crta Can Ruti, Cami de les Escoles s/n 08916 Badalona, Barcelona, Spain; 5 European Bioinformatics Institute, Wellcome Trust Genome Campus, Hinxton, Cambridge, CB10 1SD, UK

## Abstract

**Background:**

Gene expression studies greatly contribute to our understanding of complex relationships in gene regulatory networks. However, the complexity of array design, production and manipulations are limiting factors, affecting data quality. The use of customized DNA microarrays improves overall data quality in many situations, however, only if for these specifically designed microarrays analysis tools are available.

**Results:**

The IronChip Evaluation Package (ICEP) is a collection of Perl utilities and an easy to use data evaluation pipeline for the analysis of microarray data with a focus on data quality of custom-designed microarrays. The package has been developed for the statistical and bioinformatical analysis of the custom cDNA microarray IronChip but can be easily adapted for other cDNA or oligonucleotide-based designed microarray platforms. ICEP uses decision tree-based algorithms to assign quality flags and performs robust analysis based on chip design properties regarding multiple repetitions, ratio cut-off, background and negative controls.

**Conclusions:**

ICEP is a stand-alone Windows application to obtain optimal data quality from custom-designed microarrays and is freely available here (see "Additional Files" section) and at: http://www.alice-dsl.net/evgeniy.vainshtein/ICEP/

## Background

DNA microarrays are a popular high throughput technique to perform genome-wide molecular and genetic experiments. The method requires computational post-processing of primary data, which can be a challenging task due to high data variability. Variability results from each of the many steps involved in such an experiment [1]. Computational data processing is required for image processing, extraction of raw data, storage and normalization of the raw data, feature extraction, final data analysis and biological interpretation of the results. Several software packages are available to perform the described tasks but it is frequently necessary to develop custom-made solutions to fulfil individual requirements and appropriate statistical evaluation of gene array data [2,3].

Experiments on whole-genome arrays, such as Affymetrix GeneChips are highly reproducible, but still are unable to provide reliable data for all genes, especially for those with low expression levels. Moreover, there are technical limitations on array design. For example, standard glass slide arrays can not accommodate more than 60,000 spots, including all controls and replicates. This is often not sufficient for complex genomes such as the human genome. The relationship between fluorescent signal intensity and gene expression levels is linear only for a certain range of concentrations of spotted material. Thus differences in the linearity range can become more pronounced for larger whole-genome arrays [4]. In contrast, customized microarrays contain a selection of genes. This allows to include more replicates and controls. Smaller gene numbers, and higher numbers of repetitions will increase reliability of the data obtained from each microarray experiment, especially for those genes that are expressed to low levels.

IronChip is a cDNA microarray platform specifically designed to analyze genes related to iron metabolism. We have developed two types of this platform to analyze both human and mouse genes [5,6]. The design of this microarray enables detection of small but physiologically significant changes in gene expression, due to the high number of repetitive features. The current version of the mouse IronChip contains 520 genes involved in iron homeostasis and related pathways. To improve array sensitivity and data robustness, each gene on the array is represented by several ESTs. Each EST, in turn, is represented by a minimum of six spots. Some of the most relevant iron-related genes are represented by up to 24 spots. This microarray further contains a collection of negative controls, specificity controls and positive (spike-in) controls [6]. Custom microarray platforms, such as the IronChip, provide more data than required or exploited in standard statistical analysis. To incorporate all the advantages such a chip design offers for data analysis we developed the IronChip Evaluation Package (ICEP). ICEP makes use of the high number of repetitions to improve data quality. The comparison of different ESTs enables reliable detection of transcript-specific regulation (e.g. alternative splicing variants of the same gene). Analyses of the positive and negative controls allow precise calculation of a reliable ratio cut-off as well as to estimate background noise, respectively.

## Implementation

**Figure 1:**
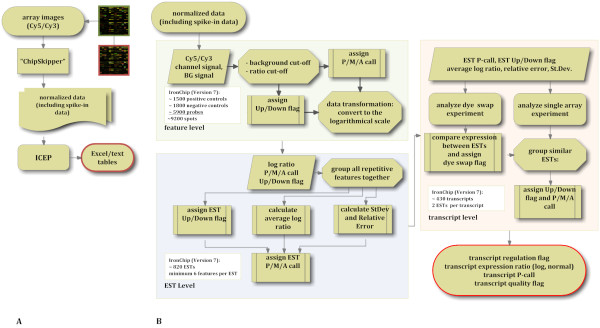
**IronChip analysis work flow**. (A) Flow chart of ICEP data processing and evaluation. Data evaluation with ICEP is organized into three functional modules: Single feature, EST and transcript evaluation. (B) In our application example, hybridized microarrays were scanned on a GenePix 4000B Microarray Scanner (Axon Instruments, Union City, CA, USA) and processed (feature background subtraction and normalization) by the ChipSkipper software [8]. ICEP uses these output files (generic tab-delimited text tables containing the normalized signal intensity and background data) for further analysis.

ICEP exploits a collection of Perl programs and utilities with a Perl Tk GUI (graphical user interface). The Perl routines were all newly custom written for the purpose of rapid and solid microarray data analysis. ICEP features a decision-tree based algorithm to optimize spot selection and exploit here in particular multiple repetitions of ESTs. ICEP applies grouping rules in its decision tree algorithm to calculate signal intensity ratios for each individual group of ESTs representing the same transcript (this is explained step by step in the application of the analysis pipeline together with supporting online material http://www.alice-dsl.net/evgeniy.vainshtein/ICEP/ICEP_manual.html). The pipeline is summarized in Figure 1A. ICEP does not use or require any existing software libraries and it can directly process simple tab delimited tables of array data of any type. It adds its optimized spot selection, filtering and normalization procedure to standard software such as Bioconductor [3] and can used in combination with these or equally well alone.

ICEP is packaged to a Windows executable with a PDK (Perl developer kit). It can be operated in a command line mode or in a batch mode for the analysis of multiple arrays. A simple editor allows to specify all microarray data files for batch analysis. In a command line mode the ICEP analysis core itself can be executed under any operating system supporting Perl. Some Windows specific features, such as exporting of output data to an Excel table or Perl Tk GUI requires adaptation to a specific operating system. In general, the modular structure of ICEP allows porting it to any other operating systems supporting Perl.

### Data formats

ICEP recognizes any generic tab-delimited text tables from any type of gene microarray containing the normalized signal intensities and background data (e.g. from the ChipSkipper application; [7]). Chip-Skipper generates a tab-delimited text table containing raw and normalized signal intensity values, background signal intensity, physical coordinates of a spot relative to an upper left corner of a glass slide, cDNA sample position on original PCR spotting plate (row, column, plate Nr), some flags and statistical values related to the spot geometry and other internal values. The ICEP uses only few of those columns: spot and clone coordinates, comments and background-compensated and normalized signal intensity value from both channels. The build-in utility recognizes not only different formats of a Chip-Skipper output file, but any generic tab-delimited text file gene expression array data can be processed by ICEP using the provided flexible configuration tool and alternative input file formats are added using the provided flexible configuration tool. Results are saved in tab-delimited format or Microsoft Office Excel formats.

### Performance

**Figure 2:**
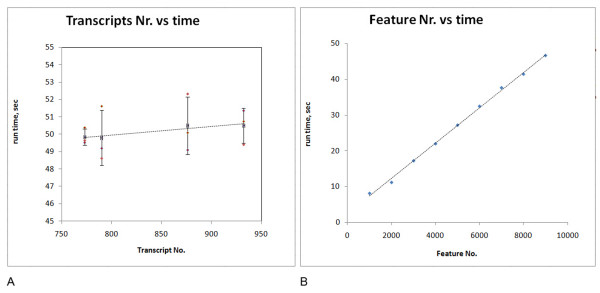
**ICEP run time chart**. (A) Run time analysis of different microarray versions containing an increasing number of features. The plot shows the resulting increase in ICEP run time for different IronChip versions. (B) A set of virtual arrays of 1000 to 9000 features was analyzed. We used a general tab delimited format. Robust statistical analysis included analysis of background noise, ratio cut-off, evaluation of multiple repetitions, detailed feature extraction and grouping results. ICEP Run time increases linearly with the increase of the total number of analyzed features. On average, ICEP evaluates 208 features per second.

We tested ICEP performance by measuring time consumption to analyze microarray data from different mouse IronChip versions (version 2.0 contains 559 transcripts, while version 7.0 contains 932 transcripts) (Figure 2A) or by analyzing a set of virtual arrays (1000 to 9000 features, with 1000 features step). On average, ICEP could evaluate 208 features per second. The time per run increases linearly with an increasing number of analyzed features (Figure 2B).

## Results and Discussion

### User interface

ICEP has been developed as a stand-alone application and does not require any special environment. It runs under any Windows operating system (it was tested on Windows 2000, XP and Vista). The interface has been designed to be highly user-friendly and interactive. It helps the user to apply all analyses while hiding the complexity of the underlying statistical methods. The interface provides easy access to different layers of microarray analysis, including single array analysis, dye-swap analysis and the generation of a final report. A step by step user manual is available in additional file 1 and at: http://www.alice-dsl.net/evgeniy.vainshtein/ICEP/ICEP_download.html

### Application of the analysis pipeline

The ICEP data analysis package was designed to be both highly flexible and user friendly. Data analysis involves an analysis pipeline that is composed of three elements: single array analysis, dye-swap experiment analysis and final report generation (Figure 1B). In addition, the pipeline can either be executed directly or in a batch analysis mode.

In our application example, we utilized the mouse IronChip microarray that contains 520 genes as well as positive and negative controls. The controls are represented by 1400 spike-in control spots (positive controls) and 2400 background control spots (negative and specificity controls). 520 genes are represented by 880 ESTs. Genes are represented by 2 or more ESTs. Each EST was spotted on the array at least 6 times. 5400 spots in total are located on the array.

At the first level of analysis (single feature level), ICEP performs logarithmical transformation of the data and separates background and control spots from the rest of the data. ICEP then calculates a background cut-off value based on median signal intensities of all background and negative control spots, and an intensity ratio cut-off value, based on signals from the spike-in controls. Intensity ratios of all remaining genes are calculated as well. At the same time, ICEP performs a feature extraction procedure, whereby all repetitive features, representing the same EST are grouped together. After calculating the background and ratio cut-off values ICEP assigns the following flags: (1) the P-call flag (true positive call), which is based on a comparison of a signal intensity of each channel with the background cut-off value (Table 1); (2) the regulation flag (significant difference in gene expression between two channels), which is based on comparison of a signal ratio to the ratio cut-off value (Table 2). At the second level of analysis (EST level) ICEP assigns further flags to ESTs and estimates the data quality based on flags recorded on a single feature level. EST P-call flags are calculated by ICEP according to the rules given in Table 3. Definition of a P-call flag (at the EST level) is based on the P-calls of individual features. Corresponding threshold is set to 60%, due to the fact that a control microarray experiment (hybridization of a Hemin- and Desferrioxamine- treated HeLa cells) shows similar results to published data only when the EST P-call threshold is about 60%. Significant increase of the EST P-call threshold causes additional false negative results while decreasing of this value causes additional false positive results

**Table 1:** P-call definition

Feature P-call	Conditions
P	The signal intensity is higher than the background cut-off value in both channels
M	The signal intensity is higher than the background cut off value in one channel and lower in the other channel
A	The signal intensity is lower than the background cut off in both channels

Definition of a P-call flag (at the feature level) to distinguish bona fide signals from background noise

**Table 2:** Regulation flag definition

Regulation flag	Conditions
UP	Ratio between signal intensities is higher than corresponding ratio cut-off
DOWN	Ratio between signal intensities is lower than corresponding ratio cut-off
NONE	Ratio between signal intensities is in between upper and lower ratio cut-off

Definition of a regulation flag (at the EST and the transcript level) to distinguish UP-/DOWN-/NONE-regulated ESTs and transcripts from each other

**Table 3:** EST P-call definition

Rule Nr.	EST P-call	Conditions	Description
1	P	P ≥ 60% ⇒ P	If more than 60% of features representing one EST have a p-call "P" then assign an EST P-call "P"
2	M	M ≥ 60% ⇒ M	If more than 60% of features representing one EST have a p-call "M" then assign an EST P-call "M"
3	A	A ≥ 60% ⇒ A	If more than 60% of features representing one EST have a p-call "A" then assign an EST P-call "A"
4	M	not 1, 2 and 3: M + P ≥ 60% ⇒ M	If criteria 1, 2 and 3 do not apply and more than 60% of features representing one EST have the p-calls "M" and "P" than assign an EST P-call "M"
5	A	not 1, 2, 3 and 4: M + A ≥ 60% ⇒ A	If criteria 1, 2, 3 and 4 do not apply and more than 60% of features representing one EST have the p-calls "M" and "A" than assign a EST P-call "A"

Definition of a P-call flag (at the EST level) is based on the P-calls of individual features and allows to distinguish UP-/DOWN-/NONE-regulated ESTs from each other

The comparison of average or median signal intensity ratios to the previously calculated ratio cut-off value yields UP/DOWN/NONE-flags, similar to the flag calculations described above. At the EST level ICEP calculates the relative error: the ratio between the standard deviation and the average of signal intensity ratios of all features representing single ESTs. At the transcript level ICEP uses the relative error as a measure of reliability of technical and biological replicates.

Preceding transcript level analysis, ICEP analyzes whether any bias has occurred as a consequence of the dye (Cy5 or Cy3 labelled nucleotides) incorporated into hybridization probes. To avoid such dye bias in two-colour microarray hybridizations the experimental and the control sample are routinely labelled with Cy5 and Cy3 labelled nucleotides, respectively, plus the other way around (dye swap). Such analysis avoids inconsistent signal intensity ratios that are artefacts due to the dye incorporated into the hybridization probe. Depending on whether an EST shows a similar average signal intensity ratio within the dye-swap data set, ICEP defines a dye-swap reliability flag (Table 4).

**Table 4:** Dye-swap reliability flag definition

Dye-swap flag	Conditions
Absent	EST shows the p-call "A" in the Cy5 and Cy3 experiment
Non reliable	The Cy5 and the Cy3 experiment show identical regulatory behavior (both UP, both DOWN)
Non regulated	EST does not show any regulation in both experiments
TRUE	EST shows "P" or "M" p-calls and is UP-regulated in the Cy5 and DOWN-regulated in the Cy3 experiment, or vice versa.
TRUE	EST shows "P" p-call and is UP-regulated or DOWN-regulated in the, Cy5 while the Cy3 experiment shows a tendency towards the correct direction based on the ratio cut-off value, or vice versa
TRUE	EST shows "P" or "M" p-call, but both experiments show NONE-regulated with one is UP-regulated or DOWN-regulated other is NONE-regulated, a tendency of regulation towards the correct direction based on a ratio cut-off

ICEP determines a reliability flag by evaluating the P-call and regulation flags of ESTs. The reliability flag is used by ICEP to distinguish reliable from unreliable expression changes.

**Figure 3:**
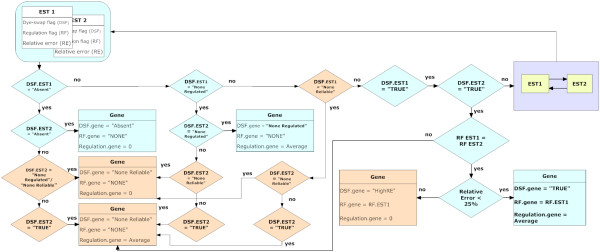
**Schema of grouping two ESTs**. The flow diagram indicates different decision steps in the analysis tree. The grouping procedure for three and more ESTs is done similarly, using rule 1 for 2 ESTs recurrently.

On the transcript level ICEP applies grouping rules to calculate signal intensity ratios for a group of ESTs representing the same transcript. For this purpose ICEP is using all quality flags described before. On this level ICEP decides whether to average signal intensity ratios from different ESTs to a single value, to treat each EST as a separate transcript or to mark the complete set of ESTs as non-reliable. ICEP is able to analyze and group values from up to six similar ESTs representing a single gene (six is the maximum value in the current version of the IronChip microarray). To illustrate the grouping procedure a scheme is presented in Figure 3 which is based on 2 ESTs representing a single gene. Table 5 represents the possible flag combinations.

**Table 5:** ESTs grouping rules (2 ESTs grouping)

EST 1		EST 2		Rel. Error	Transcript		
**DS flag**	**Regulation**	**DS flag**	**Regulation**		**Flag**	**Value**	**Regulation**

absent	N.A.	absent	N.A.		absent	0	NONE
		non regulated	NONE		non reliable	0	NONE
		non reliable	N.A.		non reliable	0	NONE
		TRUE	UP/DOWN		non reliable	Average	NONE

non regulated	NONE	non regulated	NONE		non regulated	Average	NONE
		non reliable	N.A.		non reliable	0	NONE
		TRUE	UP/DOWN		non reliable	Average	NONE

non reliable	N.A.	non reliable	N.A.		non reliable	0	NONE
		TRUE	UP/DOWN		non reliable	Average	NONE

TRUE	UP	TRUE	UP	≥15	non reliable	Average	UP
			UP	≤15	TRUE	Average	UP
		TRUE	DOWN		non reliable	Average	NONE

TRUE	DOWN	TRUE	DOWN	≥15	non reliable	Average	DOWN
			DOWN	≤15	TRUE	Average	DOWN
			UP		non reliable	Average	NONE

The EST grouping rules represent rules for a decision-tree based algorithm to summarize expression data from several ESTs, representing one transcript. These are contained within a core table.

### Validation

We applied the ICEP software to analyze IronChip microarray data. We selected a previously reported experiment that analyzes iron-loaded (hemin-treated) and iron-deficient (Desferrioxamine-treated) HeLa cells. Cellular iron overload or deficiency caused the expected changes in gene expression [6,8]. Table 6 represents the data of a dye-swap experiment. Application of ICEP reveals the previously reported and experimentally validated changes in mRNA expression of genes such as Tfrc (Transferrin receptor 1), Slc11a2 (NRAMP2; DMT1: Natural resistance-associated macrophage protein 2; Divalent metal transporter 1) and Ftl (Ferritin light chain; Ferritin L subunit).

**Table 6:** Expression increase in iron-loaded and iron-deficient cells

Gene name	Regulation (D/H)	Final flag	Ratio(av)	P-call (av)
TFRC	UP	TRUE	5, 23 ± 0, 22	P
MT2A	UP	TRUE	1, 9 ± 0, 69	P
EPAS1	UP	TRUE	1, 76 ± 0, 1	P
SLC11A2	UP	TRUE	1, 57 ± 0, 24	P
ACTB	UP	TRUE	1, 47 ± 0, 29	P
ALAS2	UP	TRUE	1, 46 ± 0, 11	P
FTL	DOWN	TRUE	-1, 58 ± 0, 06	P
HSPH1	DOWN	TRUE	-2, 28 ± 0, 13	P
HMOX1	DOWN	TRUE	-3, 17 ± 0, 16	P
HSPA1L	DOWN	TRUE	-3, 8 ± 0, 46	P
HSPA1A	DOWN	TRUE	-5, 3 ± 0, 03	P

The table represents the average ratios of differentially expressed genes in Hemin-and Desferrioxamine-treated HeLa cells. The relative errors are shown. The table contains only selected columns and genes. The full table is attached (see Additional files 1)

The complete data set (additional file 2) and ICEP installation package (additional file 3) are available in the "Additional Files" section and on-line at the web page.

### Comparison to other microarrays analysis software

We compared the performance of ICEP to other genome array tools such as Bioconductor [3], Gene-Spring (Agilent, Santa Clara, CA) [9] and a web-based tool called "Expression Profiler" [10]. While GeneSpring contains a big collection of array analysis tools it is inferior in the processing speed. Bioconductor is faster than ICEP (about 20% in running time) but as well as GeneSpring by default has limitations in processing multiple repetition features compared to ICEP. Using Bioconductor and R programming language it is possible to implement any feature recognition and statistical algorithm, but this requires additional coding work and tests. Expression profiler provides a very convenient interface and world-wide access to data analysis, but has a limited amount of statistical instruments due to its web-based nature. In general, most of the available software does not offer appropriate feature extraction from different multiple spotting regimes or recognition of feature groups. GeneSpring and Bioconductor support averaging of replicated features but do not support grouping rules for the analysis of repetitive ESTs.

## Conclusion

We introduce a new flexible microarray analysis tool named ICEP, optimized for robust statistical analysis of specialized custom cDNA or oligonucleotide microarrays. Our analysis yields values for ratio cut-off, background noise, multiple repetitions and detailed feature extraction as well as grouping rules. ICEP is easily extended to support further input and output data formats and different data transformation steps can be added. ICEP allows rapid post-processing of microarray data on a user-friendly platform. Software, example data and a tutorial are open source, free and available for downloading.

## Availability and requirements

### System requirements

ICEP runs on any computer with minimum of 256 Mb of RAM and any Windows operating system.

### Availability

ICEP installation package and updates, user manual and example data set are available for downloading at http://www.alice-dsl.net/evgeniy.vainshtein/ICEP/

## Abbreviations

Cy3/Cy5: reactive water-soluble fluorescent dyes of the cyanine dye family. Cy3 dyes are yellow-orange (550 nm excitation, 570 nm emission), while Cy5 is fluorescent in the red region (650/670 nm); EST: Expressed Sequence Tags -ESTs are small pieces of DNA sequence (usually 200 to 500 nucleotides long) that are generated by sequencing either one or both ends of an expressed gene; GUI: Graphical User Interface; ICEP: IronChip Evaluation Package; PDK: Perl developer kit.

Gene names

TFRC: Transferrin receptor 1 MT2A: Metallothionein 2 EPAS1: Endothelial PAS domain-containing protein 1; Hypoxia-inducible factor 2 alpha (HIF-2 alpha) SLC11A2: Solute carrier family 11 (proton-coupled divalent metal ion transporters), member 2; Natural resistance-associated macrophage protein 2 (NRAMP2); Divalent metal transporter 1 (DMT1) ACTB: Actin, beta, cytoplasmic ALAS2: Aminolevulinic acid synthase 2, erythroid FTL: Ferritin light chain; Ferritin L subunit HSPH1: Heat shock 105 kDa/110 kDa protein 1 HMOX1: Heme oxygenase (decycling) 1 HSPA1L: Heat shock 70 kDa protein 1-like HSPA1A: Heat shock 70 kDa protein 1A.

## Authors' contributions

YV, MUM, MS, AB contributed to the conceptualization of the method. YV developed and implemented the methods, wrote the code and the manuscript. MUM, MWH, TD, AB provided substantial intellectual contribution to the manuscript. MUM and MS provided the application cases. MUM and MWH initiated, supervised, and directed the whole project. All authors read and approved the final manuscript.

## Funding

This work was supported by the Bundesministeriums fur Bildung und Forschung (Hepatosys) [0313074D to TD, 0313073B to MM].

## Supplementary Material

Additional file 1 **ICEP user manual**. ICEP manual.pdf file contains a local copy of a user manual web page http://www.alice-dsl.net/evgeniy.vainshtein/ICEP/ICEP_manual.html. Click here for file

Additional file 2 **ICEP installation package**. ICEP.MSI file contains installation logic and all necessary files to install ICEP on any Windows operating system. Windows Installer is required to execute MSI package. In the case it is not present on the system, one could download it from Microsoft web page. Click here for file

Additional file 3 **Example dataset and ICEP analysis results**. This ZIP archive includes several files containing *a) *4 input data tables, corresponding to 2 IronChip hybridization experiments with a dye swap; *b) *2 intermediate results files with single array evaluations; and *c) *Excel file with final results. Click here for file
