# Supplementary material for: The IronChip evaluation package: a package of perl modules for robust analysis of custom microarrays
Source: BMC Bioinformatics. 2010 Mar 1;11:112. doi: 10.1186/1471-2105-11-112 (PMC2838865; doi:10.1186/1471-2105-11-112)
Supplement: Additional file 1 — ICEP user manual. ICEP manual.pdf file contains a local copy of a user manual web page http://www.alice-dsl.net/evgeniy.vainshtein/ICEP/ICEP_manual.html. [file 1471-2105-11-112-S1.PDF]

IronChip Evaluation Package (ICEP)

About ICEP

introduction

user interface

User Manual

Supplementary figures

Downloads

Contact

Links

Introduction

The IronChip Evaluation Package (ICEP) is a collection of Perl utilities and an easy to use data evaluation pipeline for the analysis of microarray data. The package has been developed for the statistical and bioinformatical analysis of the custom cDNA microarray IronChip but can be easily adapted for other cDNA or oligonucleotide-based microarray platforms of similar design. ICEP is using decision tree-based algorithms to assign quality flags and performs robust analysis based on the chip design properties regarding multiple repetitions, ratio cut-off, background and negative controls.

top

User interface

The ICEP package is designed for post-processing of the IronChip microarray data. It consists of several utilities to operate IronChip data, including single chip analysis and batch processing.

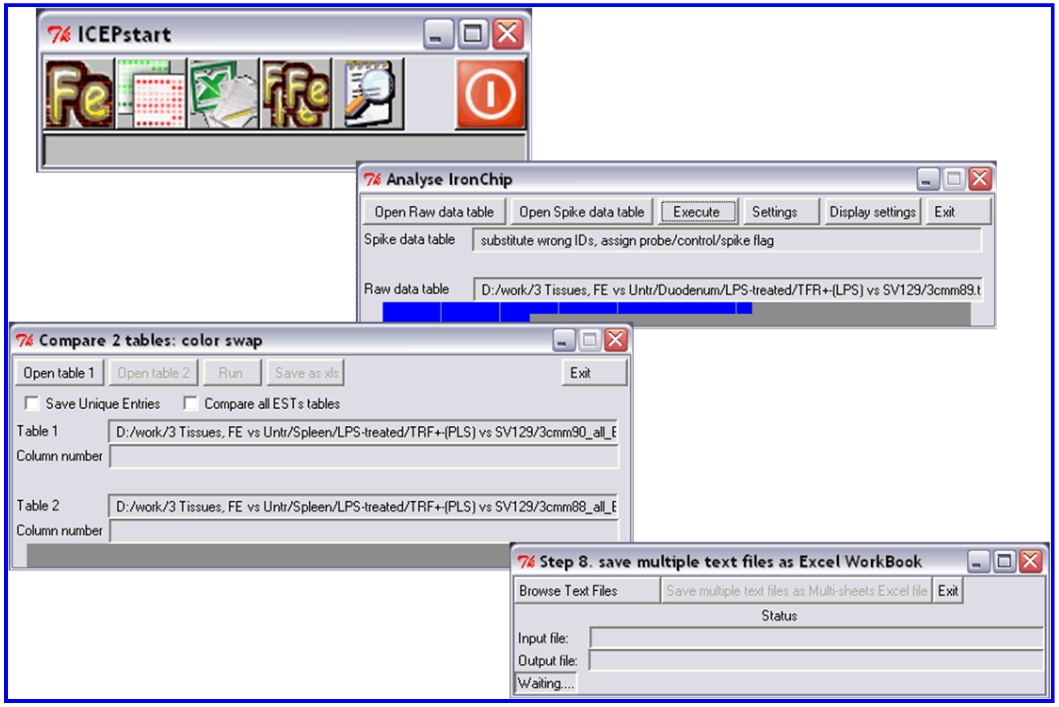

Every utility can be operated both via user friendly graphical interface, generated with perl-Tk,

and using command line.

top

©Copyright European Molecular Biology Laboratory 2009.

ICEP ****Disclaimer.***

Support: ***Yevhen Vainshtein***

IronChip Evaluation Package (ICEP)

About ICEP

User Manual

System Requirements

installing ICEP

Package content

Start using ICEP

ICEP start pannel

Single array mode

Analysis settings

Batch analysis

Batch editor

Analyse Color Swap

Convert text to Excel

Example data set

Generic microarrays

Supplementary figures

Downloads

Contact

Links

System requirments:

- Any version of MS Windows (testet on Windows 2000, XP, Vista)
- Minimum 256 MB RAM (recommended 512 MB)

Installing ICEP

- download ICEP from the **Downloads** page
- After downloading "ICEP.msi", start installation (by duble-clicking on ICEP.msi) and follow on-screen instructions.
- If required, download as well example data set to the separate folder
- Start ICEP using shortcut on the desktop

Windows Vista users: please install ICEP to alternative location (not to the default c:\program files \ folder). With standard sequrity settings, Windows Vista do not allow ICEP to modify own configuration files (batch settings and array analysis settings)

top

Package content

ICEP consist of 5 major utilities:

- ICEP start pannel (ICEPstart.exe)
- Single array analysis (Analyse_IronChip.exe)
- Color swap analysis (Analyse_ColorSwap.exe)
- Batch mode starter (ICEPbatch.exe)
- Convert multiple text files to Excel workbook (Text2xls.exe)

Each utility can be run separatly without ICEP start pannel.

top

Start using ICEP

- Prepare array data

- Analyse (feature background subtraction and normalization) by the **ChipSkipper** images derived from Cy3 and Cy5 channels of an IronChip microarray.

- Create a separate folder for an experiment. Figure below represents typical folders structure for color swap experiment with several tissues and treatments:

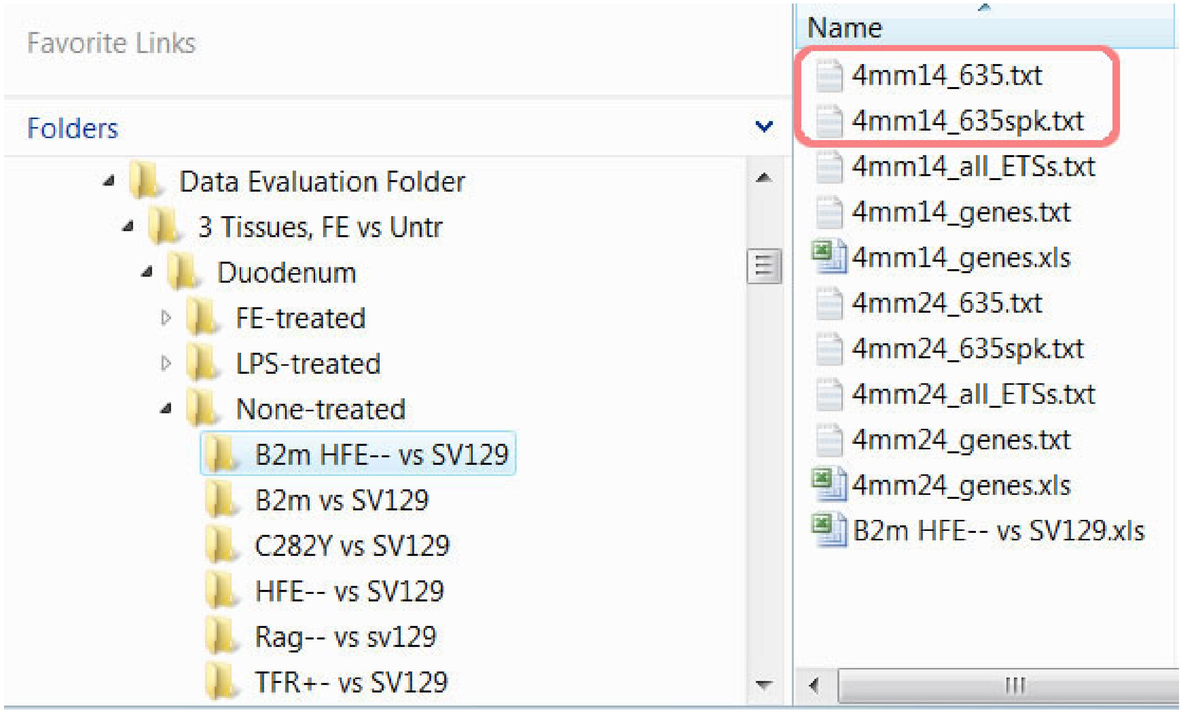

On the right pannel, two files "4mm14_635.txt" and "4mm14_635spk.txt" is a gloabl-normalized data and spike-normalized data derived from hybridisation of an murine IronChip (batch 4mm). Such two files (normalized and spike-normalized) are required to start analysis with the ICEP.

- Start ICEP with a shortcut 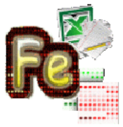 on the desktop

top

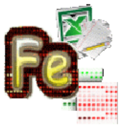 **ICEP start pannel**

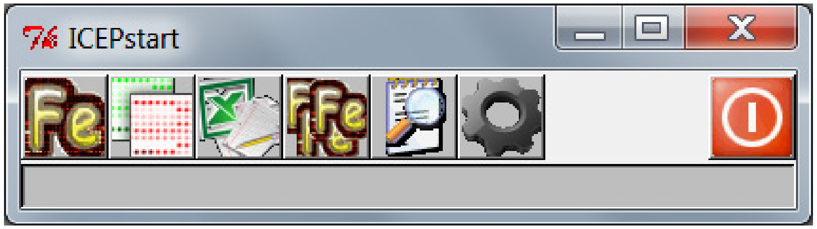

Start pannel contains buttons, allowing to start correponding utilitis. Table below contains a description:

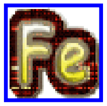	**Single array analysis**
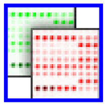	**Color swap analysis**
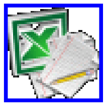	**Convert multiple text files to Excel workbook**
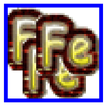	**Batch mode starter**
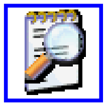	**Batch mode editor shortcut**
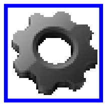	**Change ICEP analysis settings**

top

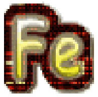 Single array analysis

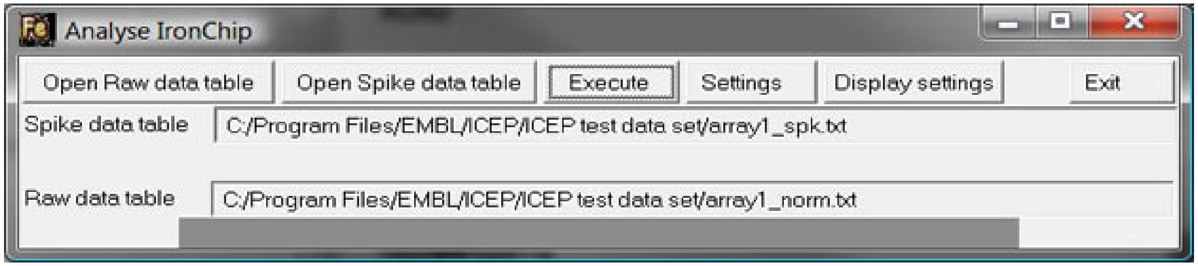

Single array analysis application (Analyse_IronChip.exe - AIC) is a major application of the ICEP. It allows:

- to perform analysis of a single array (by default IronChip),
- view and change analysis setting both for single and batch mode

Application has a very simple user interface. One can load gloabl-normalized ("Open Raw data table" button) and spike-normalized ("Open Spike data table" button) data files generated by ChipSkipper, start analysis ("Execute" button), change analysis settings ("Settings" button) and view current analysis settings ("Display settings" button).

Start array analysis:

- load gloabl-normalized file
- load spike-normalized file
- Press "Execute" button to start analysis

top

ICEP analysis settings

"Settings" button activates the following window:

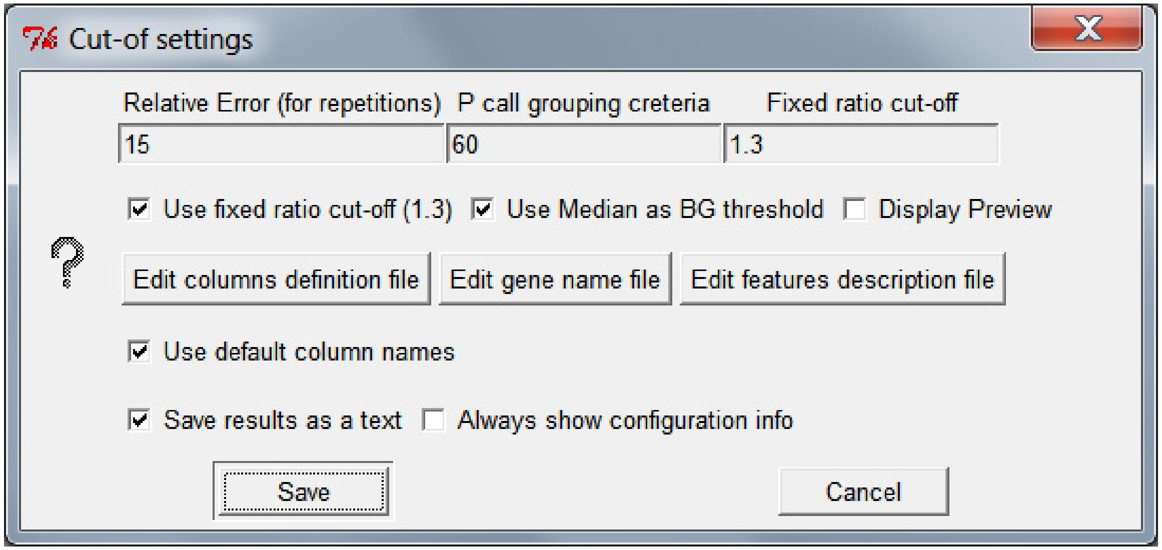

Below is a description of all analysis options:

Field name	Default	Description

Relative error	15	Relative error treshold determines stringency of a filtering according to a spread of a signal intensity values of repetetive featureas from the average. It can go from 1 to 100.<br><br>Smaller value of relative error treshold makes filtering conditions more stringent.
P call grouping creteria	60	Each EST on the array represented by 6 to 24 features. By teqnical resons, some features could hybridise less efficiently than others or could be contaminated.<br><br>P-call grouping creteria determines how many dublicated faetures (in % from total number of replicates) should share similar P-call.<br><br>Bigger value of a P-call treshold makes filtering more stringent
Fixed ratio cut-off	1.3	Single feature is marked as regulated if the ratio of signal intensity between channels is bigger than the pre-defined ratio cut-off. For IronChip this value could be very low (1.3) because of special design and high quality
Use fixed ratio cut-off	false	**true** - use fixed ratio cut-off (1.3 by default)<br><br>**false** - calculate ratio cut-off using signals from spiked-in controls

Use median as BG treshold	false	**true** - use *median* of signal intensities of empty/buffer spotted features for background signal compensation<br><br>**false** - use *average* of signal intensities of empty/buffer spotted features for background signal compensation
Display preview	false	**true** - display preview of a original data files.<br><br>**false** - do not display preview
Use default column names	true	**true** - use default column names specified in the file "Default_IronChip_columns_configuration.txt"<br><br>**false** - use customized column definitions specified in the file "Custom_columns_configuration.txt"<br><br>(for detailes see section **"Analysis of a generic microarrays"**)
Save results as a text	true	**true** - AIC creates 3 output files: Excel workbook, containing all statistcs, relevant to this analysis, original data, filtered data and results of single array analysis; Text file containing intermediate results for color swap analysis; Text file with results of single array analysis;<br><br>**false** - AIC creates 1 output file: Excel workbook

Always show configuration info	false	**true** - display current analysis configuration overview at each application start:<br>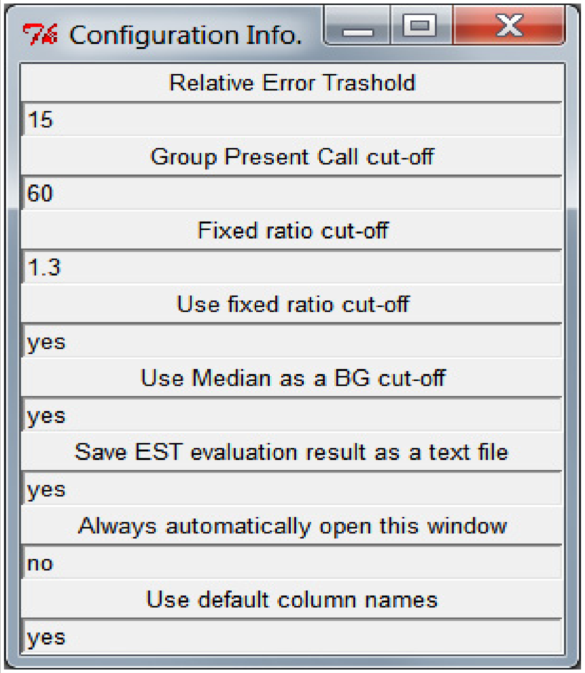
Edit columns definition file		Displays default columns definition for ICEP analysis (for detailes see section **"Analysis of a generic microarrays"**)
Edit gene name file		Displays default gene names definition file used by ICEP for feature extraction (for detailes see section **"Analysis of a generic microarrays"**)
Edit features description file		Displays default gene descriptions file used by ICEP for feature extraction (for detailes see section **"Analysis of a generic microarrays"**)

Note: settings could be modified directly, by editing configuration file "process_ironchip.ini", located in the program directory.

top

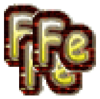 Batch analysis

Batch analysis will activates automatical array analysis mode. It does not assume any user input.

To cange analysis settings, activate single array analysis mode and press "Settings" button or modify "process_ironchip.ini file

Batch mode starter reads information about path to arrays data files from "arrays2process.txt" file. This file should be prepared in advance (see "Batch mode editor shortcut" section)

All resulting files will be saved in the source data folders.

top

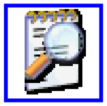 Batch mode editor shortcut

Pressing this button will pop-up default system text editor with the batch command file ("arrays2process.txt"):

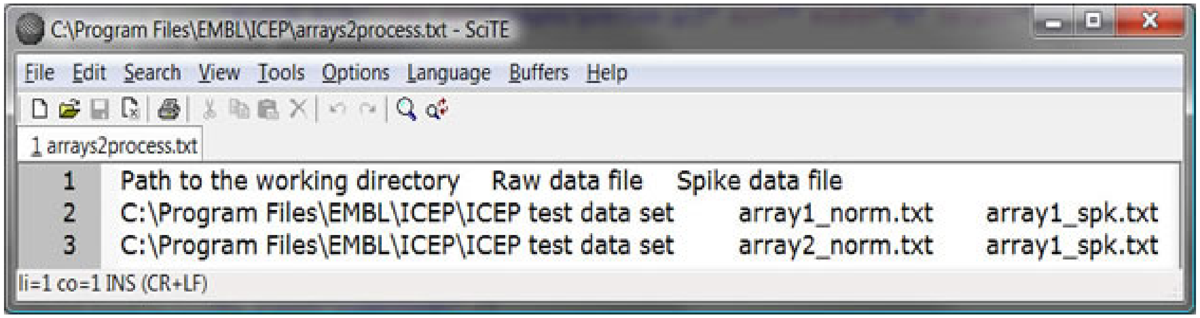

This is a tab-delimited text file with a header at the first line and information about arrays data files at the following lines. Below is a table from example data set:

Path to the working directory	Raw data file	Spike data file

C:\Program Files\EMBL\ICEP\ICEP test data set	array1_norm.txt	array1_spk.txt
C:\Program Files\EMBL\ICEP\ICEP test data set	array2_norm.txt	array1_spk.txt

Warning: in the batch analysis mode, both original data files (global-normalized and spike-normalized) from one array have to be stored in the same folder. Original data from different arrays could be stored and analysed from different folders.

top

Analysis of a color swap experiment

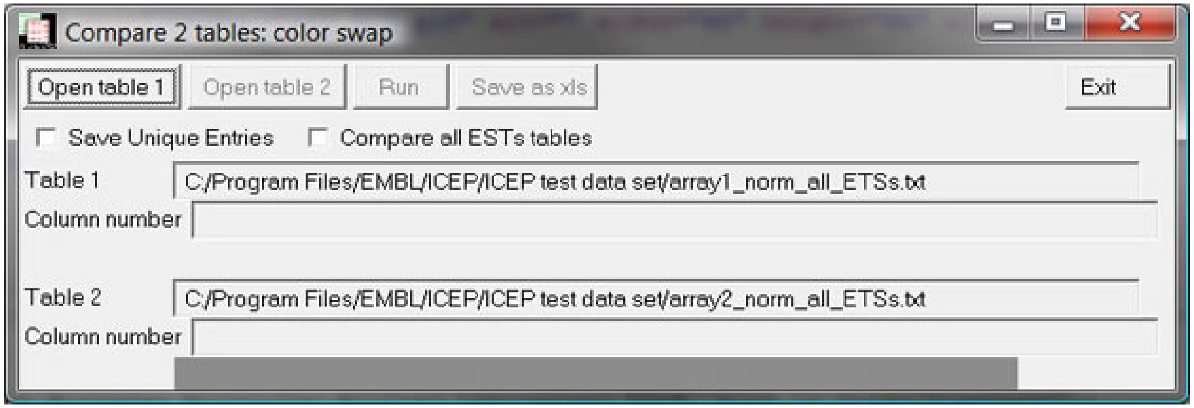

Color swap analysis application (Analyse_ColorSwap.exe - ACS) allows to compare intermediate results of single array analysis from different IronChip batches. When "Save Unique Entries" option is activated, ACS creates separate text files for ESTs present in one array batch and absent in another batch.

By default, single array analysis intermediate resulting file has a following file extention: _all_ETSs.txt

This file contains all flags and values for each ESTs on the array. Header line formaof of such file, recognised automatically.

To force ACS to recognise text file with different from the default file name, activate option "Compare all ESTs tables".

The ACS application allows as well to compare 2 text files, and identify common entries and unique entries present either in first file or in second file. This function is activated automatically, when ACS can not recognise default header line format. In this case, ACS will prompt you to select colum, containig unique ID:

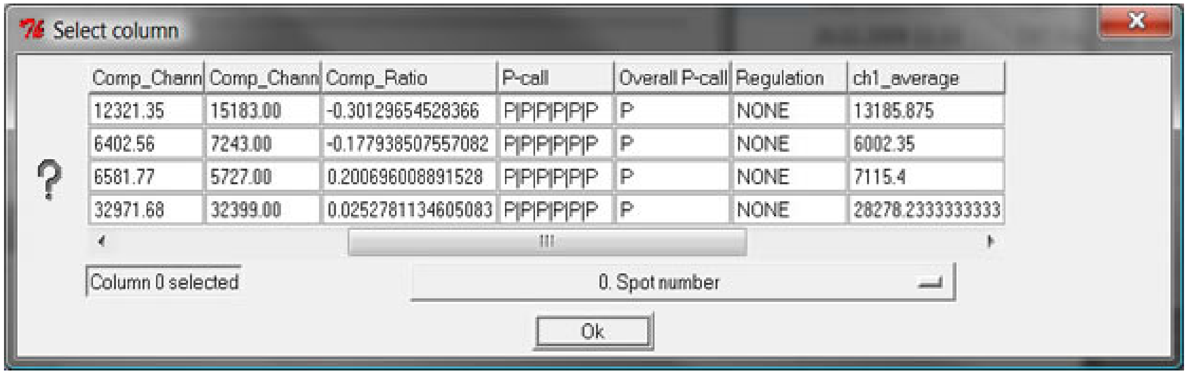

Pressing "Run" button will start either color swap analysis or table comparison. Results will be saved automatically in the same folder as a text files. To convert results to Excel, press "Save as xls" button.

top

Convert multiple text files to Excel workbook

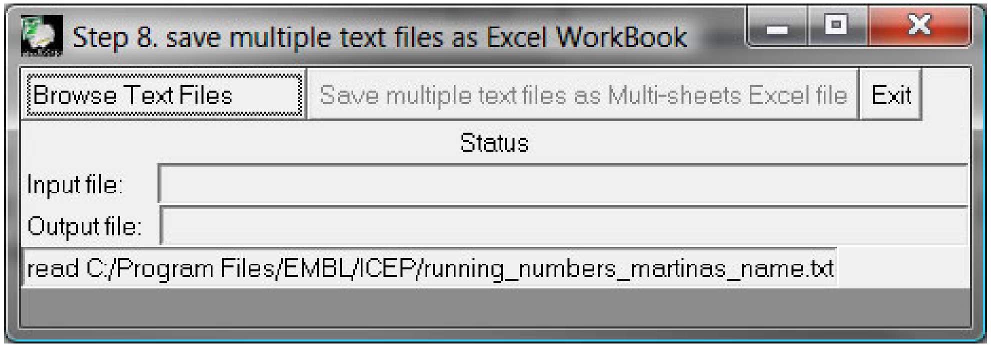

This is a stand-alown application, designed to convert multiple text files to multi-sheet Excel workbook.

"Browse Text Files" button activates a text file selection dialog:

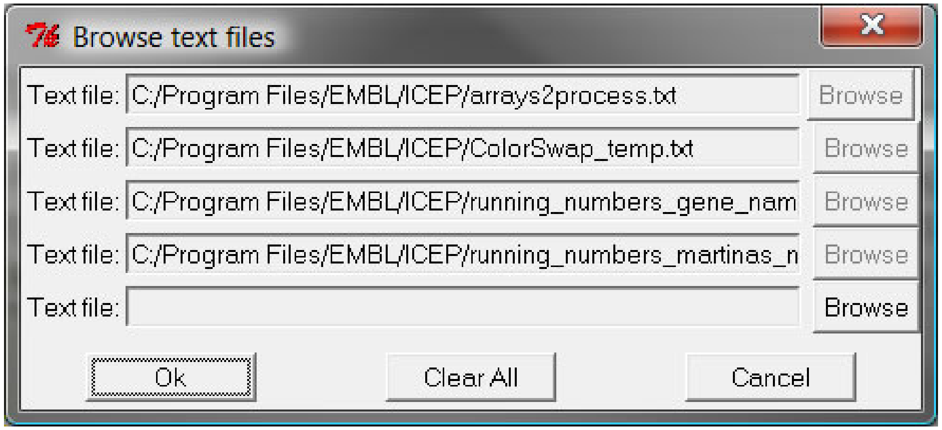

This version of text to Excel convertion utility allwos to browse manually text files from different locations and save it as a excel file.

Another automated text converter is available by request (see **Contacts**).This version can save automatically all text files from specified folder to a multi-sheet Excel file.

top

ICEP Example data set

Example data set contains 4 text files corresponding to 2 arrays, hybridized with dye swaping. Each file is a result of ChipSkipper normaliyation of raw array images.

top

Analysis of a generic microarray

ICEP recognizes any generic tab-delimited text tables from any type of gene microarray containing the normalized signal intensities and background data. The native format for ICEP is a text tab-delimited file, generated by **ChipSkipper** application.
ICEP could be conigured to work with other array types in 3 steps:

- Prepare array definition file (running_numbers_gene_name.txt)
- Prepare gene description file (running_numbers_feature_descriptions.txt)
- Prepare column definition file (Custom_columns_configuration.txt)

Deinition files located in the program folder.

Array Definition File (ADF) contains unique feature IDs, characteristic for every feature (cDNA clone, for example) spotted on the array and corresponding gene ID (like GeneBank accession number or EnsEMBL gene ID). ADF-file links internal feature IDs with corresponding genes.

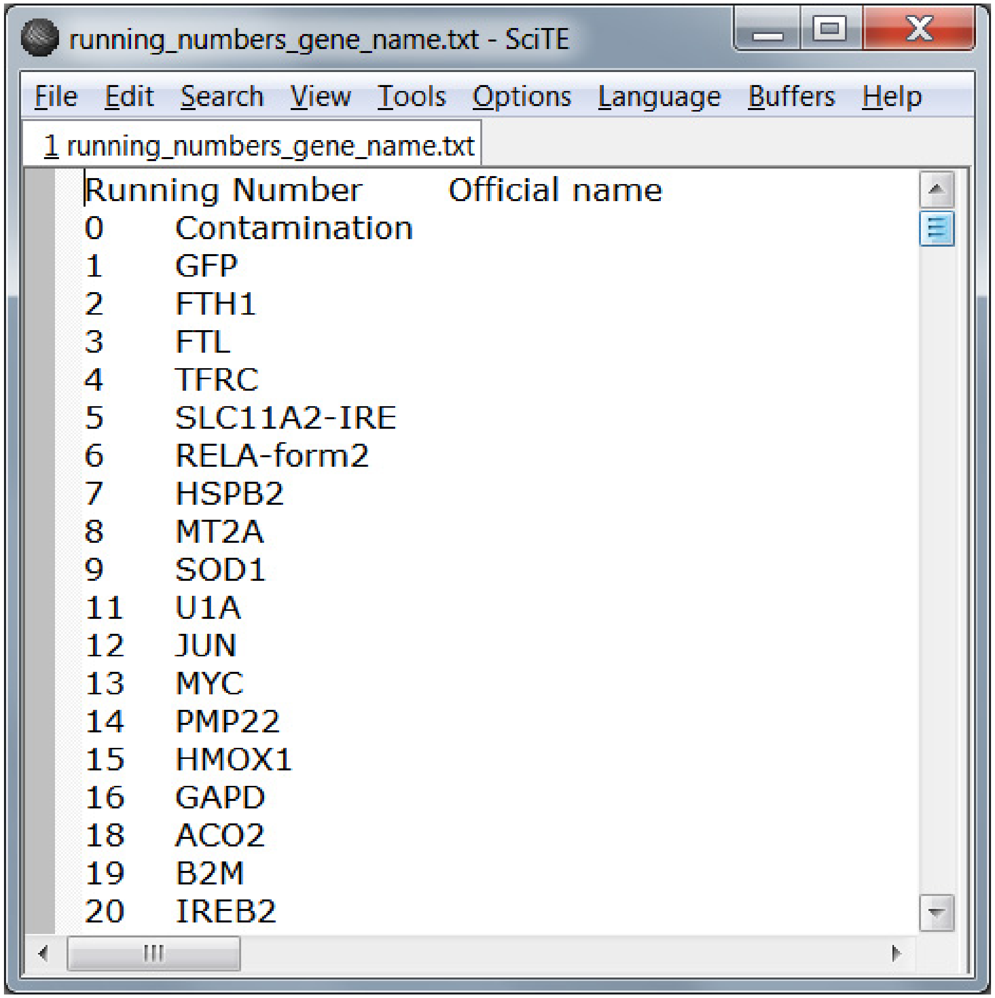

Running Number	Official name
0	Contamination
1	GFP
2	FTH1
3	FTL
4	TFRC
5	SLC11A2-IRE
6	RELA-form2
7	HSPB2
8	MT2A
9	SOD1
11	U1A
12	JUN
13	MYC
14	PMP22
15	HMOX1
16	GAPD
18	ACO2
19	B2M
20	IREB2

To edit ADF-file press [Edit gene name file] button from ICEP analysis settings dialog.

IronChip ADF file from the example above contains "Running Numbers" (cDNA Clone IDs - IronChip internal unique clone identification numbers) and Gene Names

Genes description file as well link unique feature ID (running number or IronChip) with any arbitary text entrie (for example, gene description). This table as well should be tab-delimited. To edit "genes description file" press [Edit features description file] button from ICEP analysis settings dialog.

Columns definition file allows to teach ICEP recognizing new array file firmats. To enter columns re-definition mode press [Edit columns definition file] button. This file links internal column

IDs with actual columns from input files. By default, ICEP recognize ChipSkipper output files. Columns definition file is simplified XML file:

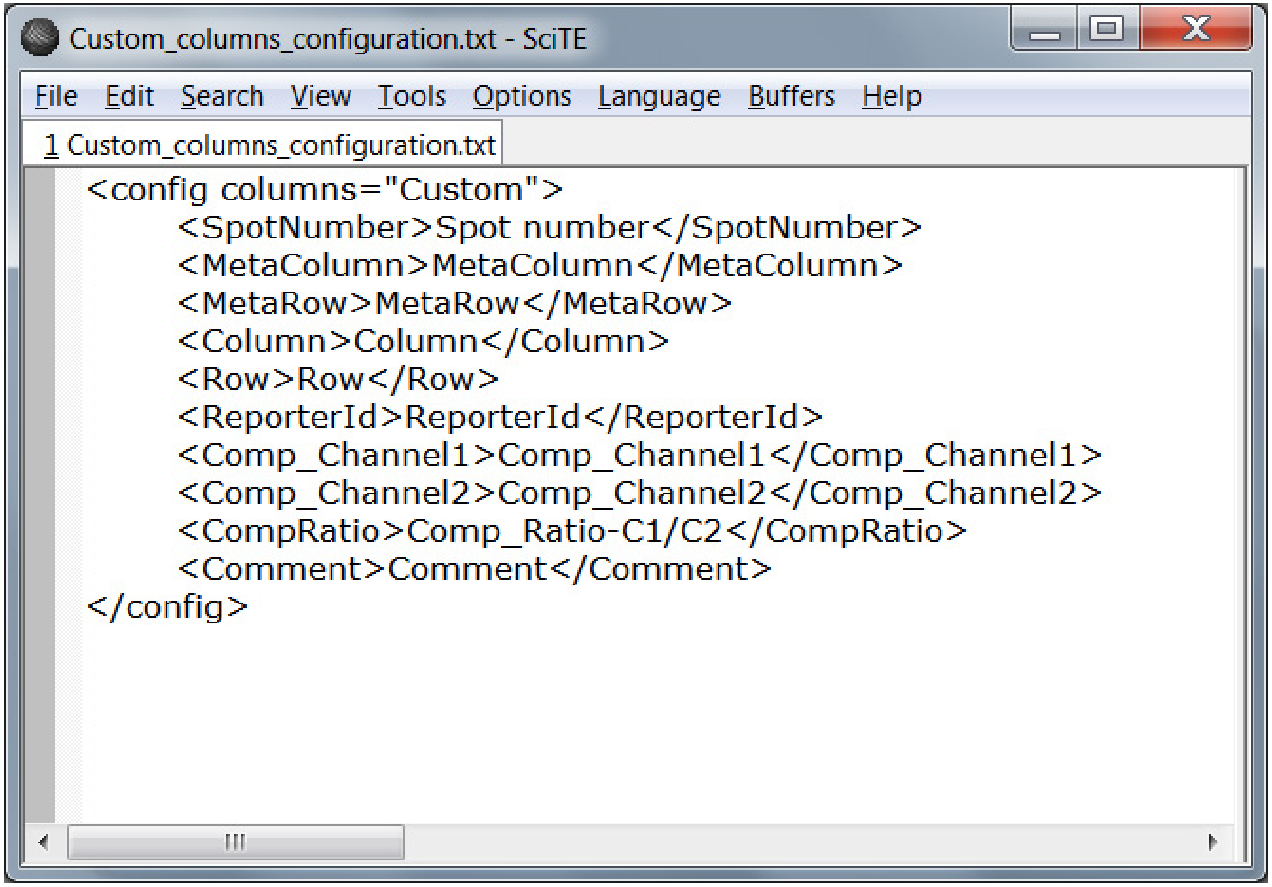


```xml
<config columns="Custom">
	<SpotNumber>Spot number</SpotNumber>
	<MetaColumn>MetaColumn</MetaColumn>
	<MetaRow>MetaRow</MetaRow>
	<Column>Column</Column>
	<Row>Row</Row>
	<ReporterId>ReporterId</ReporterId>
	<Comp_Channel1>Comp_Channel1</Comp_Channel1>
	<Comp_Channel2>Comp_Channel2</Comp_Channel2>
	<CompRatio>Comp_Ratio-C1/C2</CompRatio>
	<Comment>Comment</Comment>
</config>
```

ICEP extracts from input files only specified columns. Following columns required to run analysis correctly:

**Field name**	**Description**
SpotNumber	Number of each spot on the array (if available used for internal purposes)
MetaColumn	Spotting pin X coordinate
MetaRow	Spotting pin Y coordinate
Column	X coordinate of a feature inside a block spotted by single pin
Row	Y coordinate of a feature inside a block spotted by single pin

ReporterId	Unique Clone ID. Should contain flags for negative control/positive control/ empty spot, gene name and running number:<br>trf1/352/probe - (gene tfr1 has a unique running number 352, and this is a probe)<br>empty - empty spot<br>2xssc//negative - negative control spot<br>firluc//spike - spike-in positive control.
Comp_Channel1	Channel 1 signal intensity (local background-compensated)
Comp_Channel2	Channel 2 signal intensity (local background-compensated)
Comment	Any text comment, related to this spot

To illustrate how to define MetaColum/MetaRow/Column/Row look at the following figure:

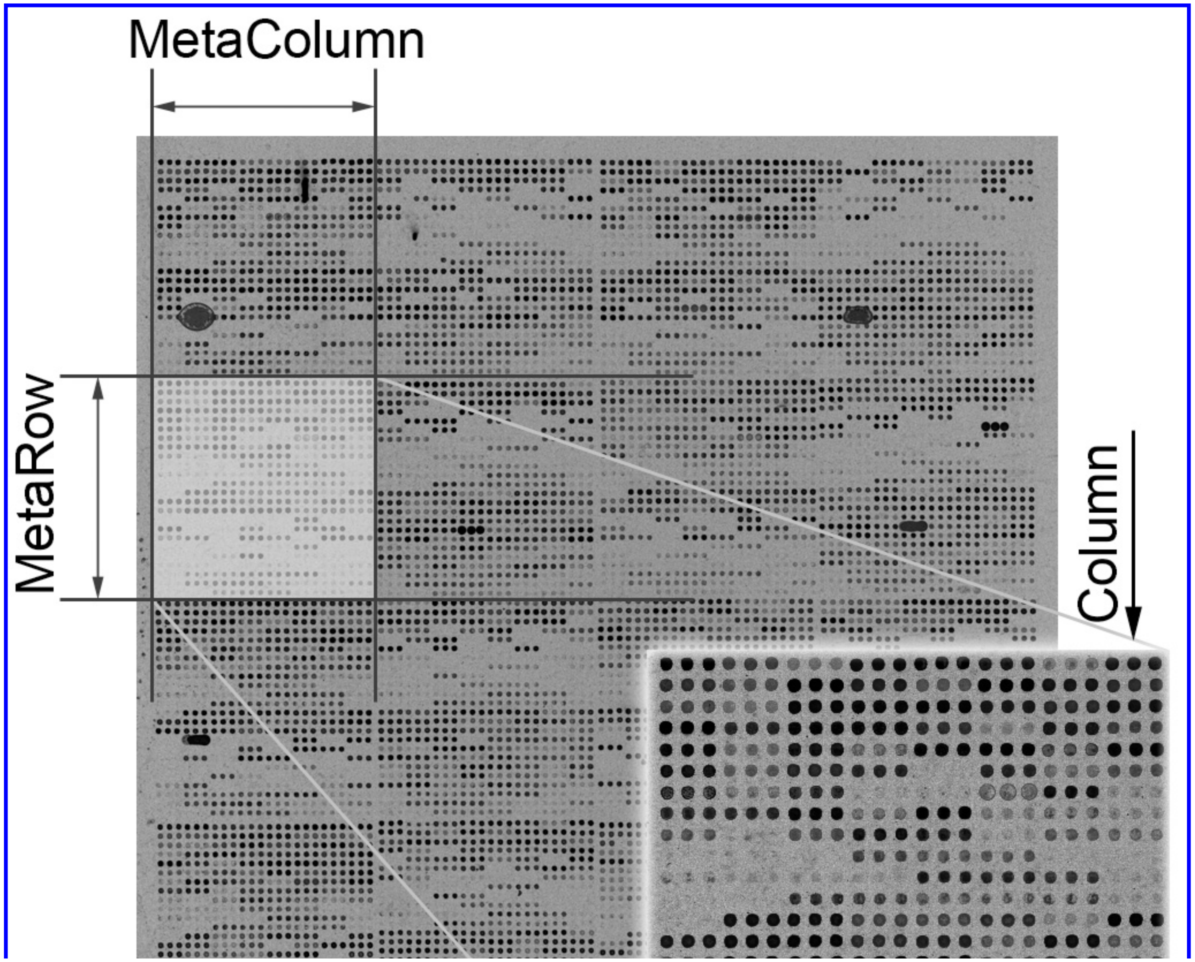

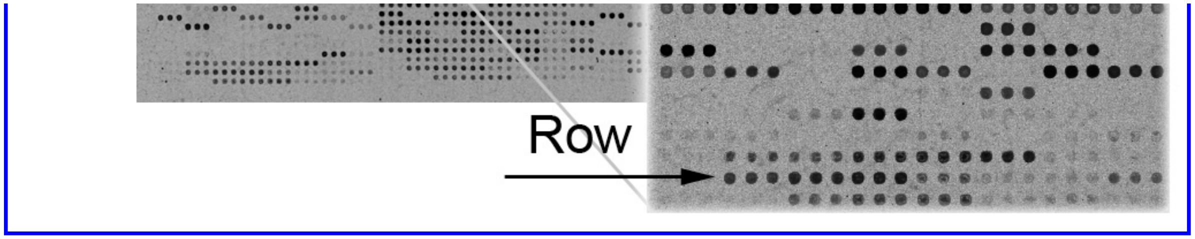

top

©Copyright European Molecular Biology Laboratory 2009.
ICEP *Disclaimer.
Support: Yevhen Vainshtein

IronChip Evaluation Package (ICEP)

About ICEP

User Manual

Supplementary figures

Figure 1

Figure 2

Figure 3

Downloads

Contact

Links

Supplementary figures

A B

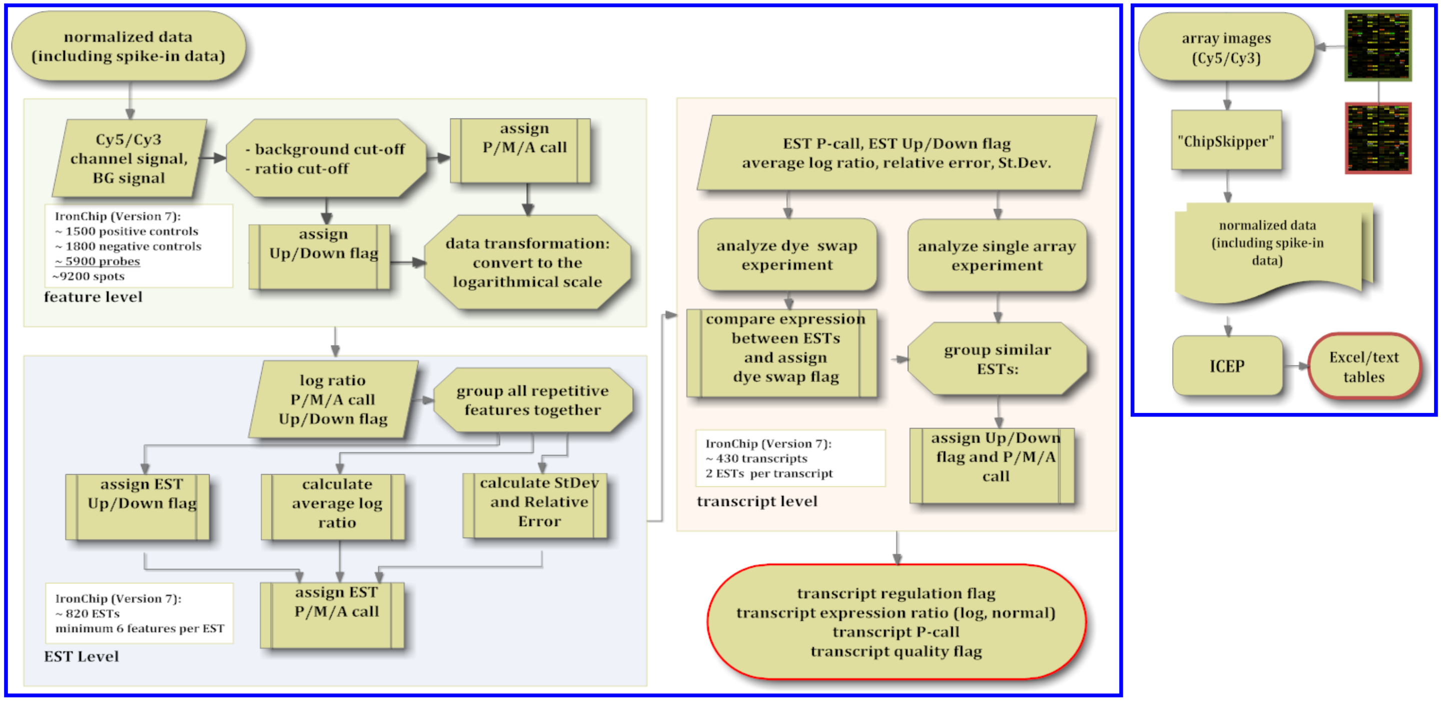

Figure 1 - IronChip analysis work flow

(A) Flow chart of ICEP data processing and evaluation. Data evaluation with ICEP is organized into three functional modules: Single feature, EST and transcript evaluation.

(B) In our application example, hybridized microarrays were scanned on a GenePix® 4000B Microarray Scanner (Axon Instruments, Union City, CA, USA) and processed (feature background subtraction and normalization) by the ChipSkipper software [8]. ICEP uses these output files (generic tab-delimited text tables containing the normalized signal intensity and background data) for further analysis.

top

A B

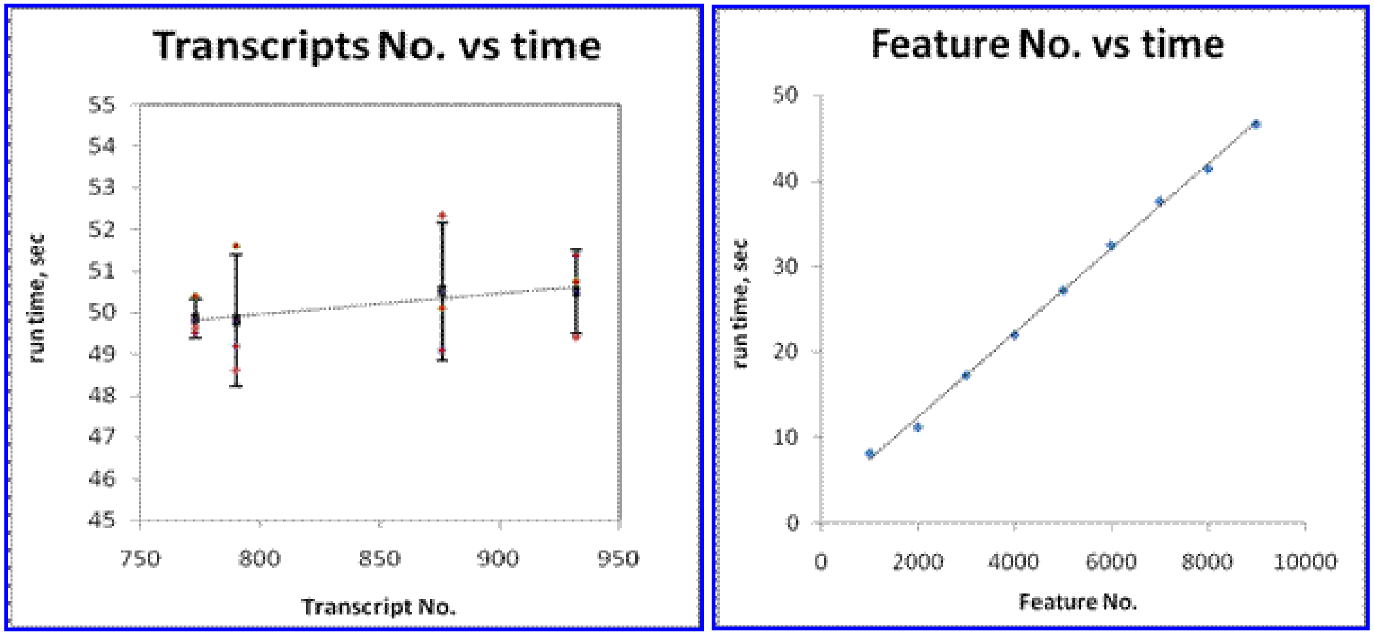

Figure 2 - ICEP run time chart

(A) Run time analysis of di erent microarray versions containing an increasing number of features. The plot shows the resulting increase in ICEP run time for di erent IronChip versions.

(B) A set of virtual arrays of 1000 to 9000 features was analyzed. We used a general tab delimited format. Robust statistical analysis included analysis of background noise, ratio cut-o , evaluation of multiple repetitions, detailed feature extraction and grouping results. ICEP Run time increases linearly with the increase of the total number of analyzed features. On average, ICEP evaluates 208 features per second.

top

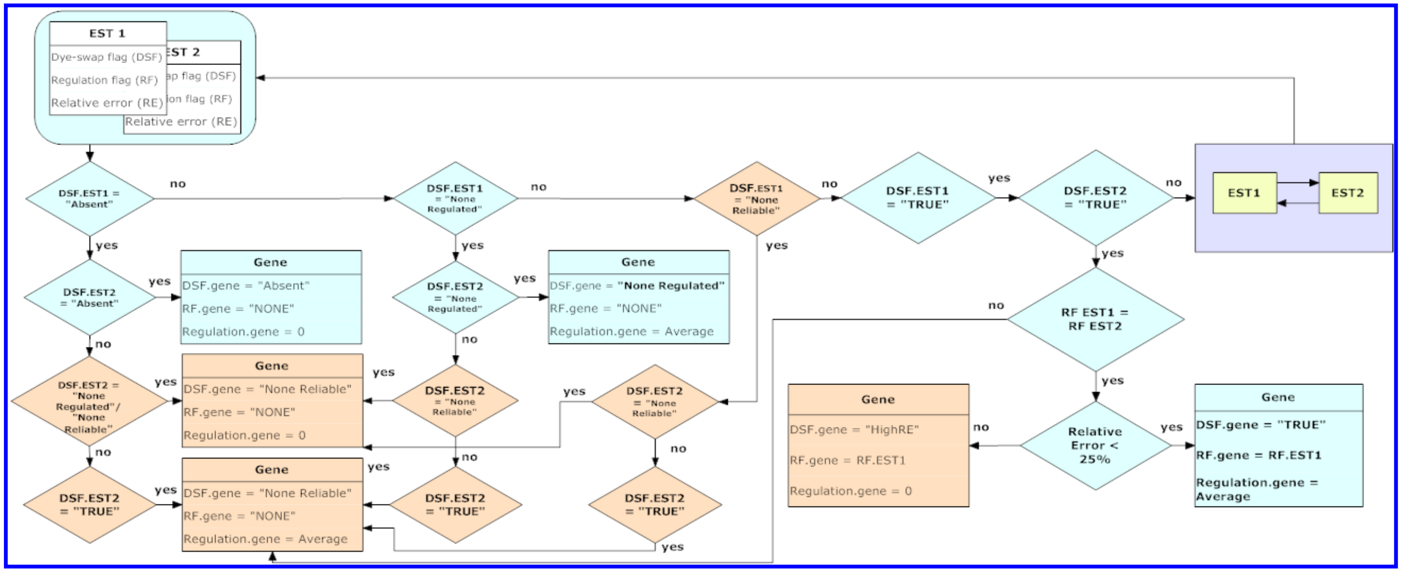

Figure 3 - Schema of grouping two ESTs

The flow diagram indicates different decision steps in the analysis tree. The grouping procedure for three and more ESTs is done similarly, using rule 1 for 2 ESTs recurrently

top

©Copyright European Molecular Biology Laboratory 2009.
ICEP ****Disclaimer.***
Support: ***Yevhen Vainshtein***

IronChip Evaluation Package (ICEP)

Download application:

Windows installation packages:

- **ICEP.msi** (11,8MB)
- **ICEP.zip** (11,5MB)

Windows version without installation:

- **ICEP_noinstall.zip** (11,4MB)
  (Unzip to a separate folder and run ICEPstart.exe to start analysis)

Download user manual:

icep.chm (411KB)

Download test set:

Original data files only: **test_data_set.zip** (3,32MB)

Original data and results: **ICEP test arrays and results.zip** (5,84MB)

Note: file "results.xls" with final result of color swap experiment was post-processed with Excel (sorting, filtering, color code, e.t.c)

top

©Copyright European Molecular Biology Laboratory 2009.
ICEP ****Disclaimer.***
Support: ***Yevhen Vainshtein***

IronChip Evaluation Package (ICEP)

With general question related to IronChip platform please contact Martina Muckenthaler: **Martina.Muckenthaler@med.uni-heidelberg.de**

With questions related to ICEP please contact Yevhen Vainshtein: **vainshte@embl.de**

©Copyright European Molecular Biology Laboratory 2009.
ICEP ****Disclaimer.***
Support: ***Yevhen Vainshtein***

IronChip Evaluation Package (ICEP)

IronChip related projects:

- IronSystems
- IronLIMS

Relative Expression Software Tool (REST)

- REST, REST-384, REST-MCS, REST-RG

- RefScout PDFManager

- Hentze Group Page
- Main Page of the EMBL-Heidelberg

top

©Copyright European Molecular Biology Laboratory 2009.

ICEP **Disclaimer.*

Support: *Yevhen Vainshtein*

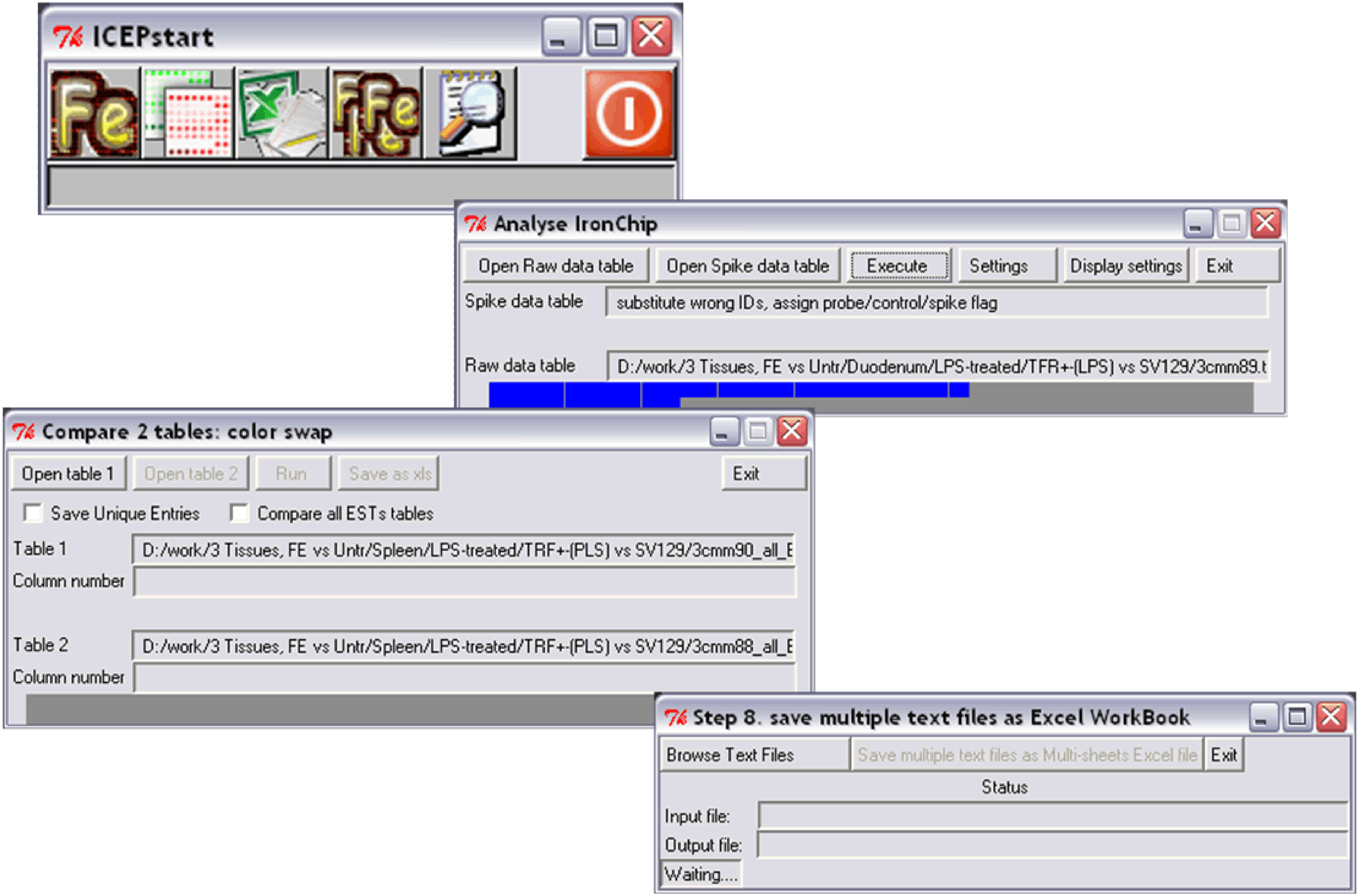
ICEPstart
Analyse IronChip
Open Raw data table
Open Spike data table
Execute
Settings
Display settings
Exit
Spike data table
substitute wrong IDs, assign probe/control/spike flag
Raw data table
D:/work/3 Tissues, FE vs Untr/Duodenum/LPS-treated/TFR+-(LPS) vs SV129/3cmm89.t
Compare 2 tables: color swap
Open table 1
Open table 2
Run
Save as xls
Exit
Save Unique Entries
Compare all ESTs tables
Table 1
D:/work/3 Tissues, FE vs Untr/Spleen/LPS-treated/TRF+-(PLS) vs SV129/3cmm90_all_E
Column number
Table 2
D:/work/3 Tissues, FE vs Untr/Spleen/LPS-treated/TRF+-(PLS) vs SV129/3cmm88_all_E
Column number
Step 8. save multiple text files as Excel WorkBook
Browse Text Files
Save multiple text files as Multi-sheets Excel file
Exit
Status
Input file:
Output file:
Waiting....

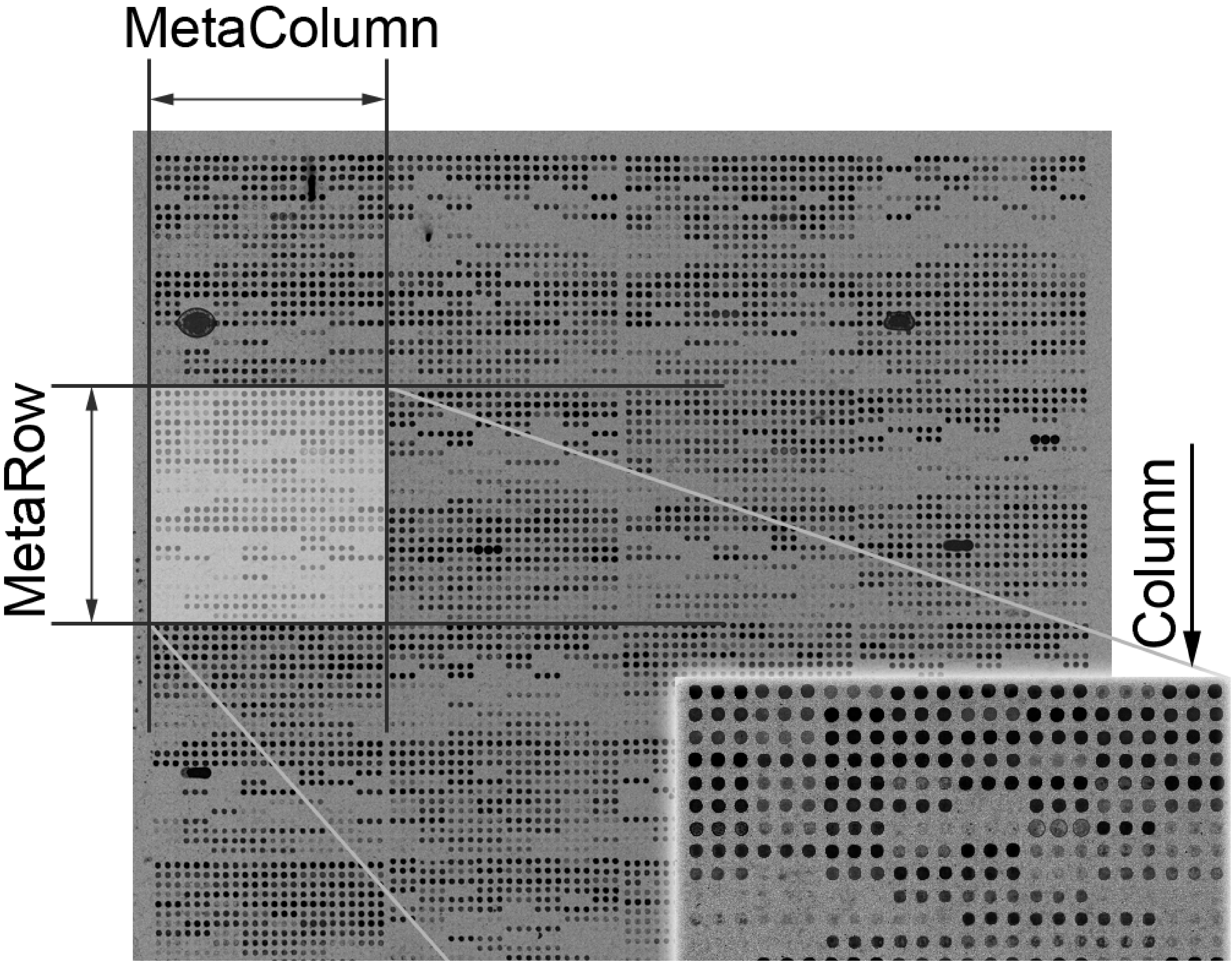
MetaColumn
MetaRow
Column

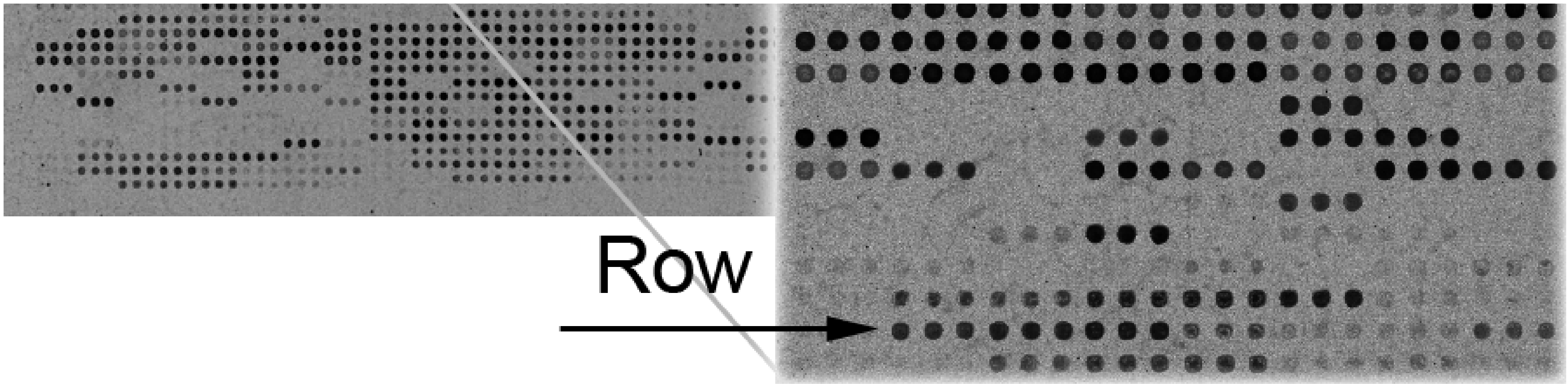
Row

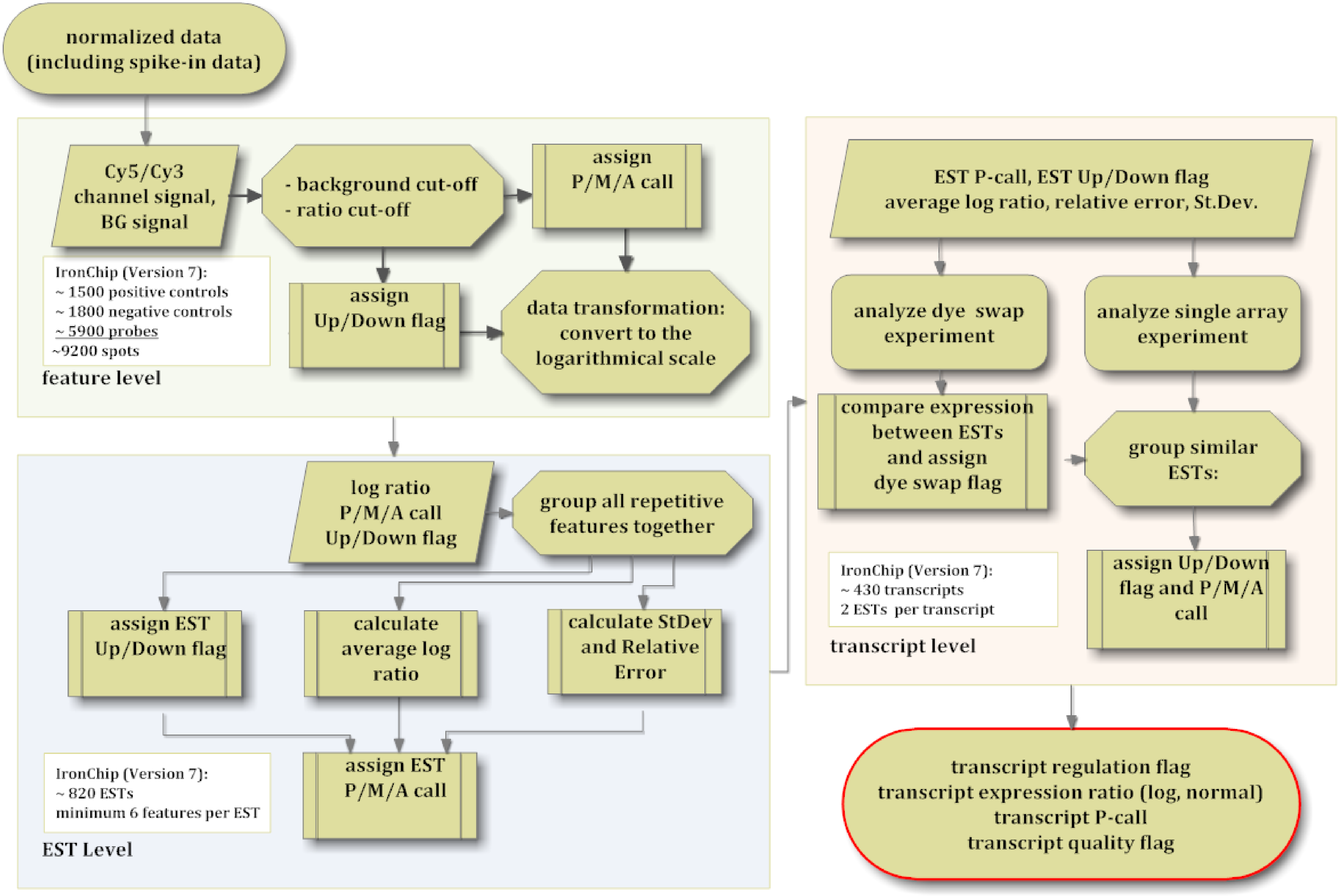
normalized data
(including spike-in data)
Cy5/Cy3
channel signal,
BG signal
- background cut-off
- ratio cut-off
assign
P/M/A call
IronChip (Version 7):
~ 1500 positive controls
~ 1800 negative controls
~ 5900 probes
~9200 spots
assign
Up/Down flag
data transformation:
convert to the
logarithmical scale
feature level
log ratio
P/M/A call
Up/Down flag
group all repetitive
features together
assign EST
Up/Down flag
calculate
average log
ratio
calculate StDev
and Relative
Error
IronChip (Version 7):
~ 820 ESTs
minimum 6 features per EST
assign EST
P/M/A call
EST Level
EST P-call, EST Up/Down flag
average log ratio, relative error, St.Dev.
analyze dye swap
experiment
analyze single array
experiment
compare expression
between ESTs
and assign
dye swap flag
group similar
ESTs:
IronChip (Version 7):
~ 430 transcripts
2 ESTs per transcript
assign Up/Down
flag and P/M/A
call
transcript level
transcript regulation flag
transcript expression ratio (log, normal)
transcript P-call
transcript quality flag

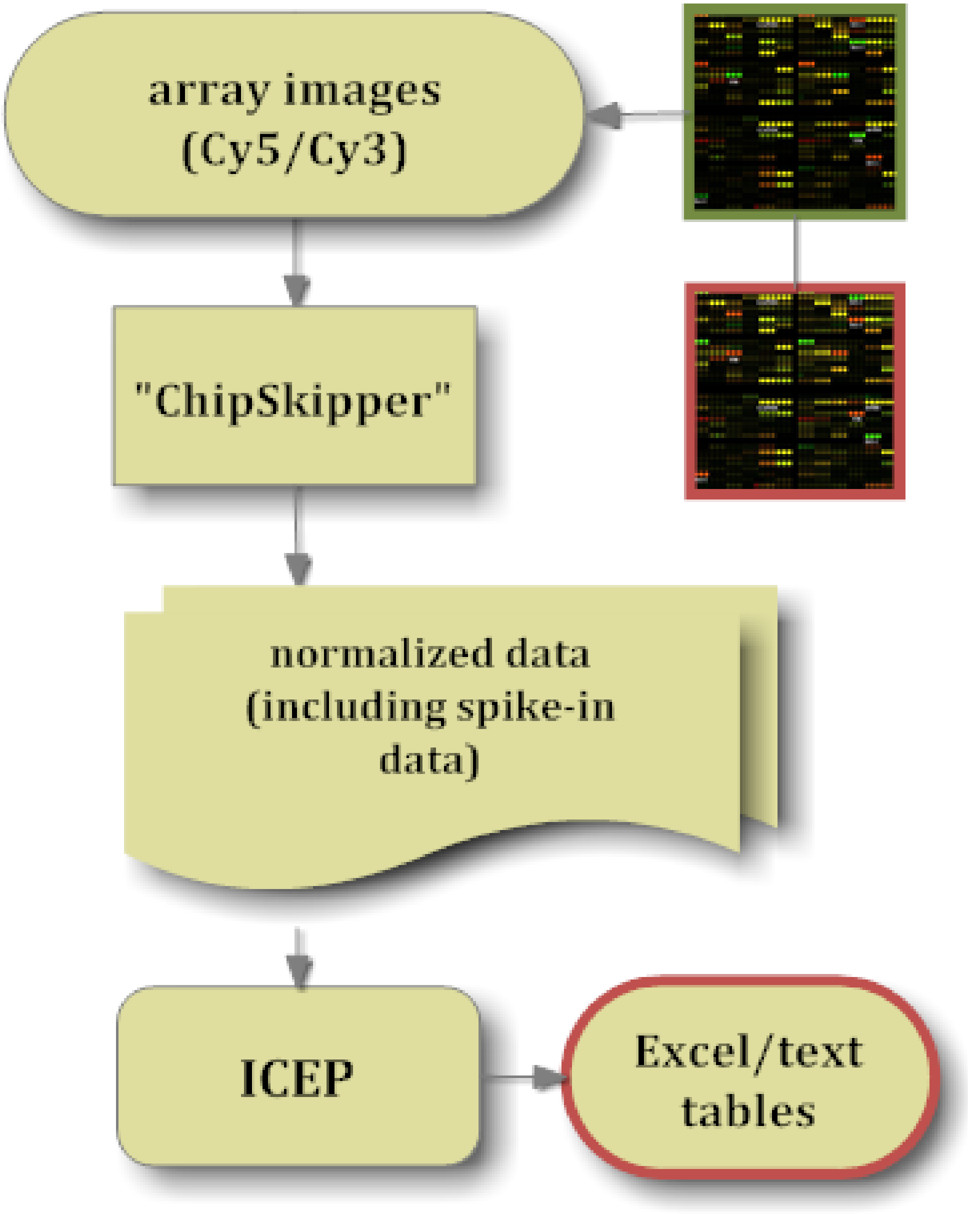
array images
(Cy5/Cy3)
"ChipSkipper"
normalized data
(including spike-in
data)
ICEP
Excel/text
tables

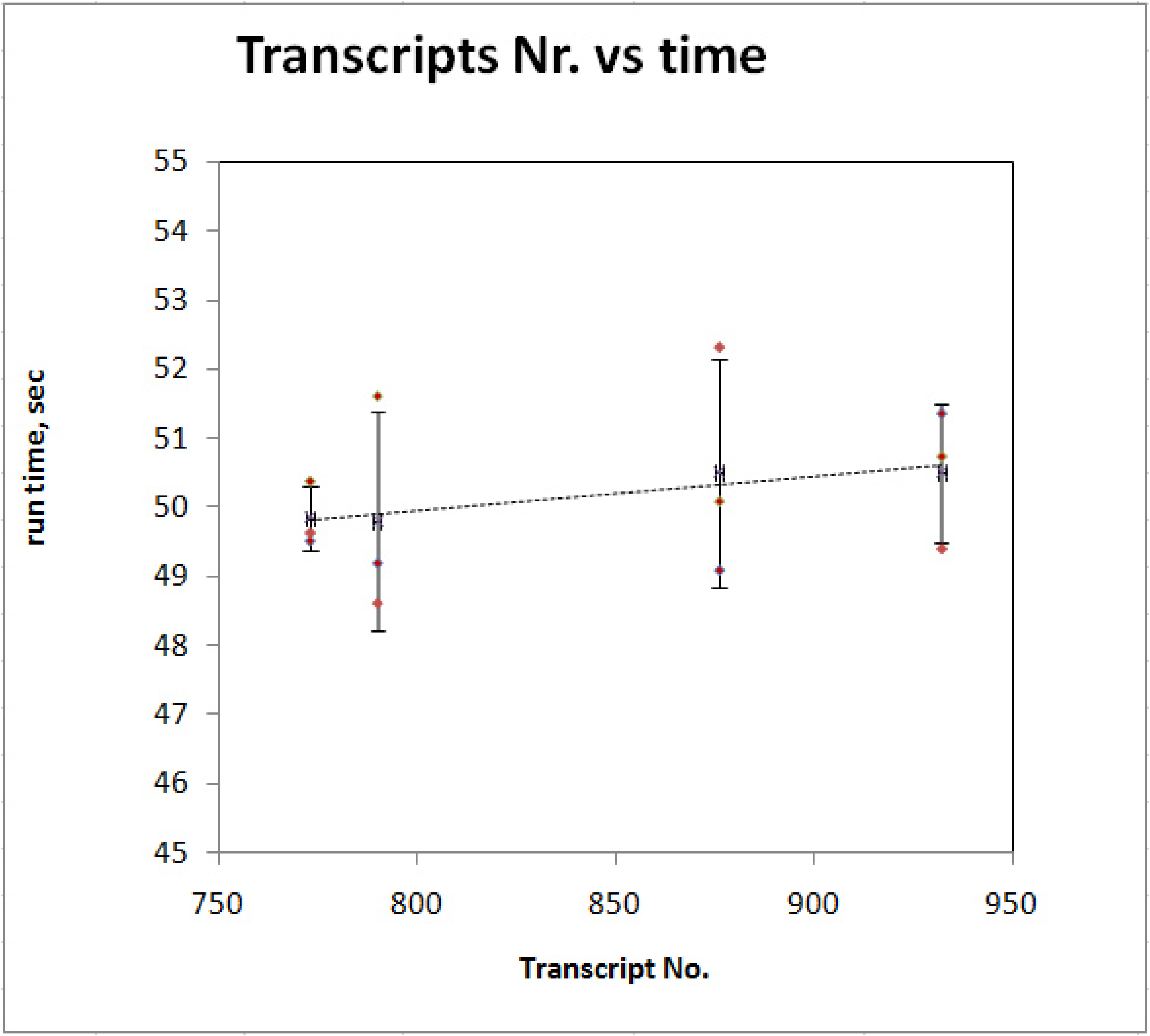
Transcripts Nr. vs time
run time, sec
55
54
53
52
51
50
49
48
47
46
45
750
800
850
900
950
Transcript No.

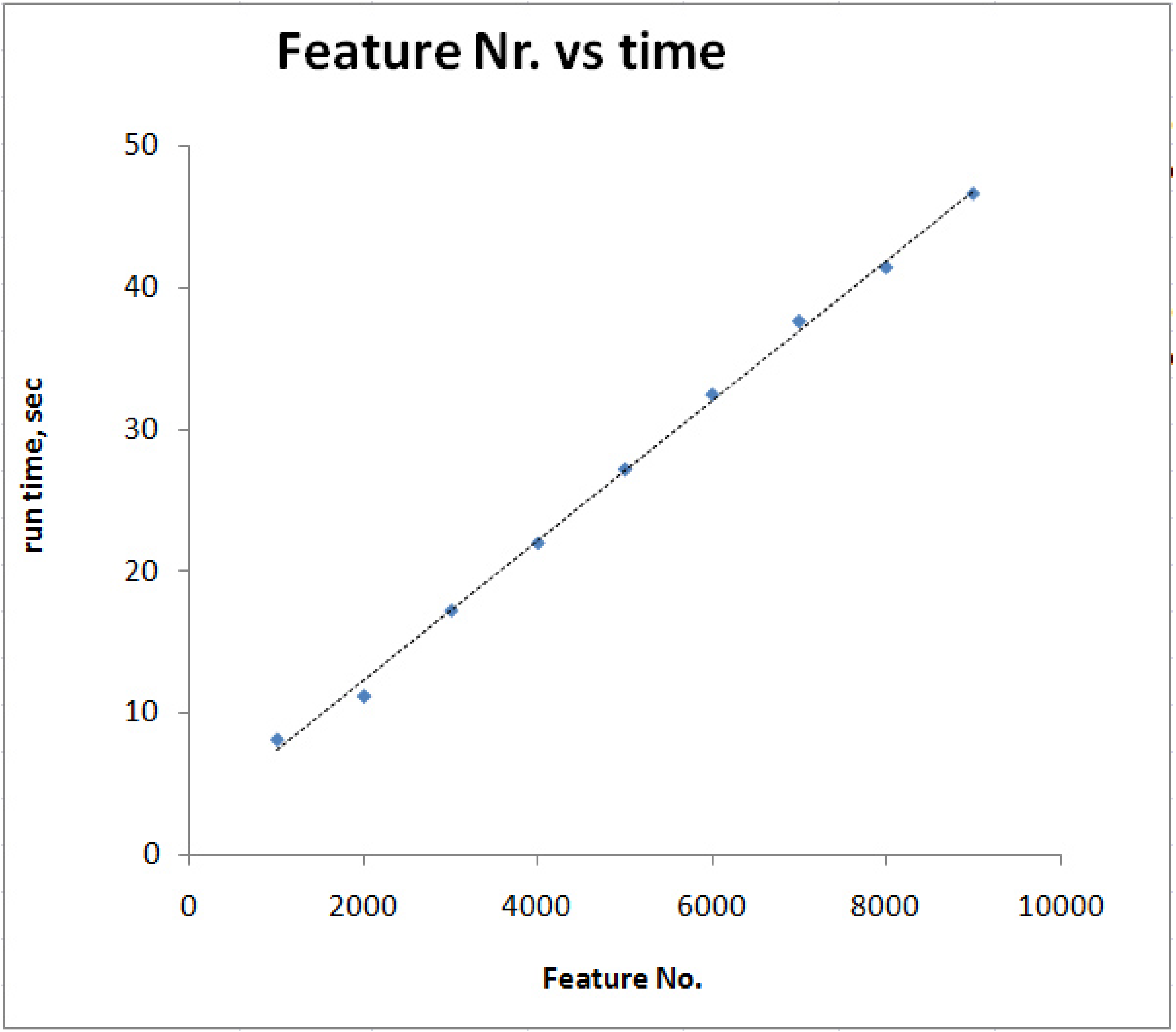

Feature Nr. vs time
run time, sec
50
40
30
20
10
0
0
2000
4000
6000
8000
10000
Feature No.

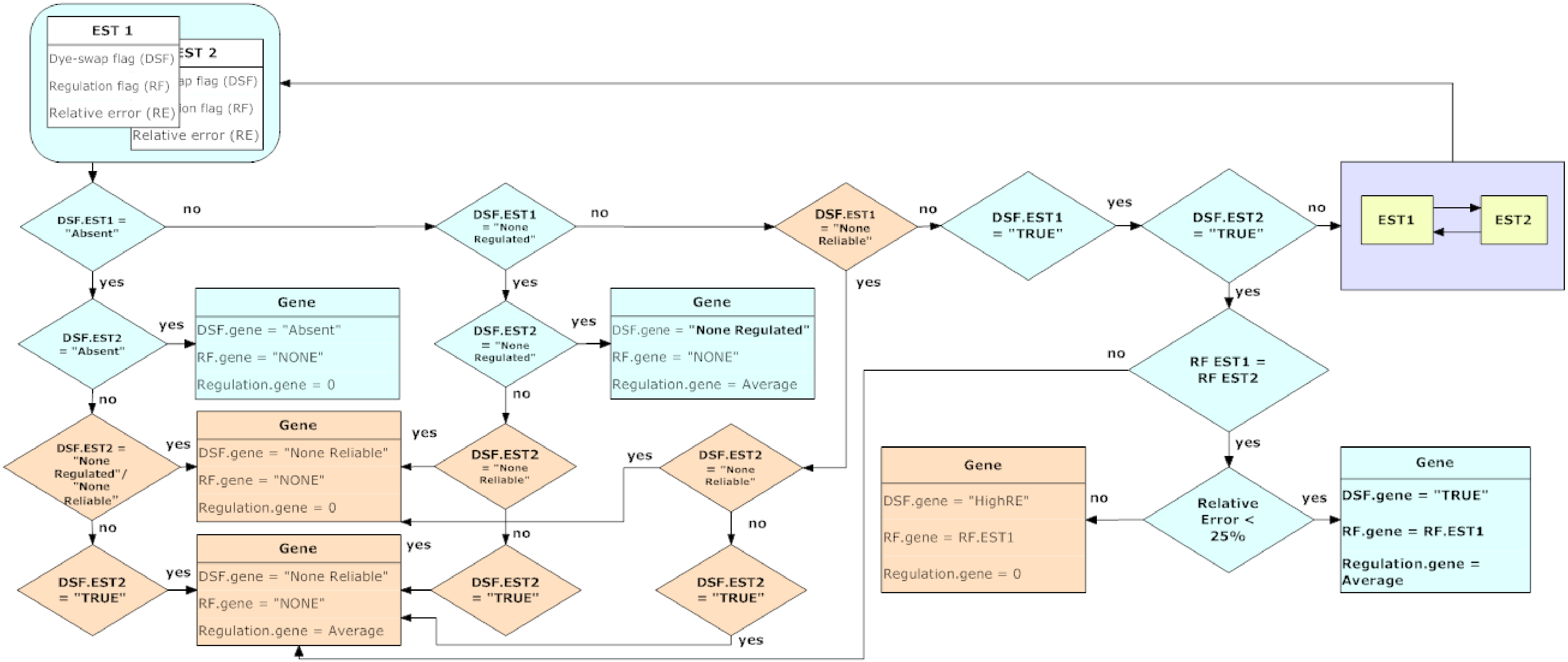
EST 1
Dye-swap flag (DSF)
Regulation flag (RF)
Relative error (RE)
EST 2
Relative error (RE)
DSF.EST1 = "Absent"
no
yes
DSF.EST2 = "Absent"
yes
Gene
DSF.gene = "Absent"
RF.gene = "NONE"
Regulation.gene = 0
DSF.EST1 = "None Regulated"
DSF.EST2 = "None Regulated"
Gene
DSF.gene = "None Regulated"
RF.gene = "NONE"
Regulation.gene = Average
DSF.EST1 = "None Reliable"
DSF.EST1 = "TRUE"
DSF.EST2 = "TRUE"
EST1
EST2
RF EST1 = RF EST2
DSF.EST2 = "None Regulated"/ "None Reliable"
Gene
DSF.gene = "None Reliable"
RF.gene = "NONE"
Regulation.gene = 0
DSF.EST2 = "None Reliable"
DSF.EST2 = "None Reliable"
Gene
DSF.gene = "HighRE"
RF.gene = RF.EST1
Regulation.gene = 0
Relative Error < 25%
Gene
DSF.gene = "TRUE"
RF.gene = RF.EST1
Regulation.gene = Average
DSF.EST2 = "TRUE"
Gene
DSF.gene = "None Reliable"
RF.gene = "NONE"
Regulation.gene = Average
DSF.EST2 = "TRUE"
DSF.EST2 = "TRUE"
